# High-sugar diet leads to loss of beneficial probiotics in housefly larvae guts

**DOI:** 10.1093/ismejo/wrae193

**Published:** 2024-10-03

**Authors:** Anna Voulgari-Kokota, Francesco Boatta, Ruud Rijkers, Bregje Wertheim, Leo W Beukeboom, Jacintha Ellers, Joana Falcao Salles

**Affiliations:** Groningen Institute for Evolutionary Life Sciences (GELIFES), University of Groningen, Nijenborgh 7, P.O. Box 11103, Groningen 9700 CC, The Netherlands; Laboratory of Microbiology, Wageningen University, Wageningen 6700 EH, The Netherlands; Amsterdam Institute for Life and Environment, Section Ecology and Evolution, Vrije Universiteit Amsterdam, Amsterdam 1081 HV, The Netherlands; Amsterdam Institute for Life and Environment, Section Ecology and Evolution, Vrije Universiteit Amsterdam, Amsterdam 1081 HV, The Netherlands; Department of Environmental Science, Stockholm University, Stockholm SE-106 91, Sweden; Groningen Institute for Evolutionary Life Sciences (GELIFES), University of Groningen, Nijenborgh 7, P.O. Box 11103, Groningen 9700 CC, The Netherlands; Groningen Institute for Evolutionary Life Sciences (GELIFES), University of Groningen, Nijenborgh 7, P.O. Box 11103, Groningen 9700 CC, The Netherlands; Laboratory of Microbiology, Wageningen University, Wageningen 6700 EH, The Netherlands; Groningen Institute for Evolutionary Life Sciences (GELIFES), University of Groningen, Nijenborgh 7, P.O. Box 11103, Groningen 9700 CC, The Netherlands

**Keywords:** housefly, microbiota, probiotics, high-sugar diet

## Abstract

The housefly (*Musca domestica*) is a common insect species with only a few recurrent bacterial taxa in its gut microbiota, because the numerous microbial acquisition routes in its septic habitats can favor transient microbes. Here, we investigated the role of the diet on the microbiota and the developmental success of a housefly strain reared on three substrates. We used a control wheat bran-based substrate, and added clotted cream and sucrose to make a high-fat, and a high-sugar substrate, respectively. The conducted survey revealed that, in contrast to the high-fat diet, the high-sugar diet caused lower developmental success and less diverse microbiota, in which several lactobacilli were replaced with *Weissella* bacterial phylotypes. Cultures with sucrose as the sole carbon source confirmed that a *Weissella confusa* strain, isolated from larvae, could utilize sucrose more efficiently than other tested lactic acid bacteria; a result also supported by gene function prediction analysis. Enhancing the rearing substrate with *Limosilactobacillus fermentum* and *Lactiplantibacillus plantarum* strains, which were isolated from control larvae, could not only revert the negative effect of the high-sucrose diet on development, but also increase the gut bacterial diversity. In our study, we show that the microbiota shifts in response to the high-sucrose diet did not benefit the host, that showed lower developmental success. In contrast, high-sucrose favored specific components of the microbiota, that continued to be enriched even after multiple generations, outcompeting beneficial bacteria. Also, microbiome manipulation showed the potential of probiotics to rescue host performance and restore the microbiome.

## Introduction

Insect physiology relies on interactions with microbes, that are associated with a wide range of host phenotypes and functions [1, 2]. The common housefly, *Musca domestica* is no exception. Although the housefly microbiota has mostly been studied due to the species’ capacity to vector pathogens [3–7], there are recent studies focusing on the species’ natural and recurrent microbiota [8–14], indicating specific bacteria as important for development and oviposition [8, 15, 16]. The facts that the species is commonly found in septic environments [17] and has recently been proposed as suitable for waste bio-remediation and protein production [18–21] make the study of its microbiota even more relevant [22, 23].

Microbial acquisition by the housefly depends on developmental stage and habitat [10, 12–14]. Diet is of pivotal importance too, as microbes can be acquired horizontally [11]. Housefly larvae need substrates with a viable microbial community to develop [8] and can grow on substrates with a wide variety in nutritional content and microbial composition [24]. Studies on *Drosophila melanogaster* have shown that diet and gut microbiota are in constant interplay regulating host metabolism [25]. In the case of the housefly, different dietary ingredients can favor certain bacterial taxa. For instance, even though houseflies can feed on various dietary sugars such as sucrose, fructose, glucose, and xylitol [26], each can have different effects, with sucrose, which is preferred by houseflies when dissolved [26], causing a less diverse microbiota in adults [27].

The influence of diet on the microbiome is not restricted to horizontal microbial acquisition and can persist through generations, as studies in other Diptera have shown [28]. Microbial communities may shift indirectly as adaptation to novel diets, because microbiomes can change more rapidly than host genomes [29]. Microbial variation can be a major adaptive trait for the host by extending host phenotypic plasticity [30]. Insects such as the housefly could serve as a model for investigating the gut bacterial microbiota dynamics, because they combine three traits: a restricted set of recurrent bacterial phylotypes, transient and diverse microbiota associated with environmental conditions, and short life cycles [10–12, 14]. Also, the dietary flexibility of the housefly, as we discussed it in the previous paragraph, enables us to study its response to dietary challenges across multiple generations.

In this project, we were interested in how exposure to unbalanced diets, particularly fat- and sugar-rich diets, can induce immediate and long-term changes. We used *M. domestica* to test the effect of larval substrates containing elevated levels of fat and sugar on the gut bacterial communities for two developmental stages and 13 generations. Our initial hypothesis was that the diet would affect the bacterial community and that the effect would be larger for larvae than for emerging adults. To test this, we reared houseflies on a control (CTR) diet, a high-sugar diet, and a high-fat diet. Additionally to monitoring diet-driven microbiota divergence, we assessed host fitness, by measuring larval biomass, pupation, and adult emergence. The combination of bacterial community monitoring and host fitness assessment allowed us to link bacterial diversity levels and certain bacterial taxa with developmental success. To address whether these associations were causative, we inoculated the experimental diets with specific bacterial strains and tested their impact on host fitness.

This study follows the intergenerational effect of diet on *M. domestica*. Here, we test the ability of the species to adapt to dietary changes and we investigate the bacterial shifts happening in the course of consecutive generations. Also, we tested whether the manipulation of the rearing substrate’s microbiota could be a natural way to replace healthy host microbiota.

## Materials and methods

### Housefly rearing and sampling

All housefly lines were founded from the same base population (sampled in 2018), provided by the University of Groningen after acclimatization for three generations at the Vrije Universiteit of Amsterdam. Five replicate housefly lines were set up for every treatment, resulting in fifteen experimental lines in total. Adult flies were kept in cages (model BD4M3030D; BugDorm) at 25°C and 60% humidity with a 12 h light/12 h dark cycle. Adults from all experimental lines were given water, sucrose diluted in water (20% w/v), and inactivated yeast *ad libitum*. Nine days after emergence, adults were provided with egg-laying substrate (450 g, 10:1 w/w wheat bran: inactivated yeast) for four hours. After collecting and weighing the eggs, replicates of 2500–3000 eggs were made and transferred to jars containing 300 g of substrate. Each experimental line was subsequently propagated with 2500–3000 eggs at each generation.

**Figure 1:**
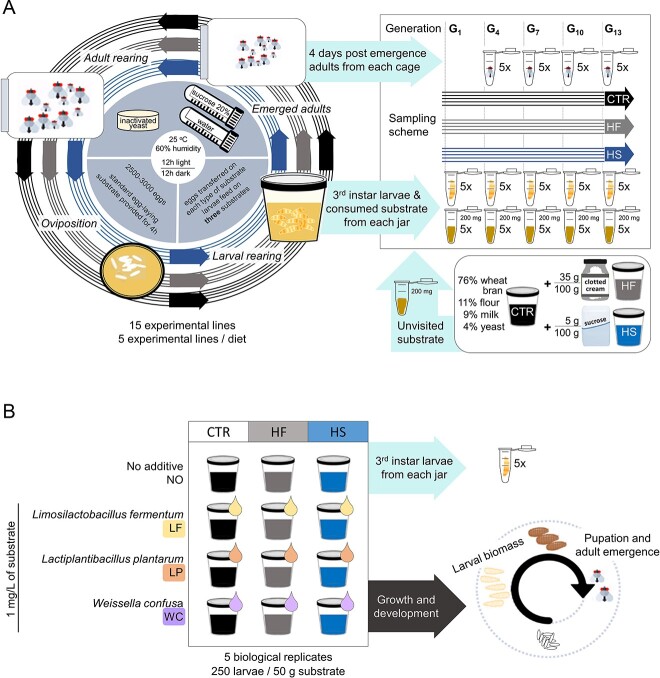
Experimental design for testing the diet effect on the housefly gut microbiota and development. We tested three larval substrates as diets: CTR stands for the control, HF stands for the high-fat, and HS stands for the high-sugar. (A) Composition of the experimental diets, schematical description of the housefly rearing and sampling scheme for the bacterial survey. For every treatment (CTR, HF, and HS), five biological replicate experimental lines were maintained. (B) the effect of probiotic inoculation was evaluated in houseflies acclimatized to the different diets. These were sampled throughout their life cycle to measure larval biomass, pupation, and adult emergence. Five biological replicates were set up for every experimental diet. Additionally, identical replicates were set up for every diet enhanced with lactic acid bacteria.

Three dietary treatments were used for the larvae: the control (CTR), the high-fat (HF), and the high-sugar diet (HS). CTR consisted of 76% w/w wheat bran, 11% w/w flour, 9% w/w milk powder, and 4% w/w inactivated yeast. HS additionally contained 5 g sucrose for every 100 g of substrate, and HF additionally contained 35 g clotted cream for every 100 g of substrate. The specific quantities of added ingredients were chosen after running pilot experiments that secured that the elevated levels of sugar and clotted cream did not cause larval mortality. We sampled larvae from generations 1, 4, 7, 10, and 13 (Fig. 1A). A sample of five 3^rd^ instar larvae was taken from every jar 4 days after eclosion and the remainder were left to pupate. After adult emergence, flies from each jar were placed in separate cages and the same process was repeated. Five adults were sampled from every cage after four days. We sampled adults from generations 4, 7, 10, and 13 (Fig. 1A).

Additionally to housefly larvae and adults, we sampled 200 mg fresh substrate in the beginning of the experiment, before using it to set up the rearing of larvae, and consumed substrate from the same jars and at the same time we sampled larvae. In total, 213 samples were taken: 75 samples from larvae, 60 from adults, 75 from consumed substrate, and three samples from fresh substrate. All samples were taken with sterile tweezers and were immediately stored at -20°C until further processing. Dissections of larval and adult guts were conducted under sterile conditions by covering each specimen with PBS to avoid desiccation. After 13 generations, one line for each of the three experimental diets was maintained for follow-up experiments.

Total genomic DNA was isolated from all gut and substrate samples using the Qiagen PowerSoil extraction kit, following the manufacturer’s instructions. A swab sample from the dissection tools and one from the kit solutions were included in DNA isolation as controls. Sequencing libraries were constructed at the University of Minnesota Genomics Center. Quality control was conducted with qPCR to estimate copy numbers for the 16S rRNA gene, using primers V4 515F and V4 806R and annealing temperature of 55°C, as previously described [31]. The library preparation included purification, normalization, and pooling. Sequencing consisted of a 2 × 300 bp MiSeq System (Illumina) run on the V4 region of the bacterial 16S rRNA gene.

### Isolation of bacteria from larvae

To isolate bacteria for supplementing larval diets, samples from larval guts of the maintained lines (>13 generations) were used for bacterial culturing in MRS medium (De Man, Rogosa, and Sharpe). Incubation was performed in broth anoxically at 32°C for 48 hours and for 48 hours more after plating on MRS agar. Picked colonies were used as templates for PCR amplification targeting the whole 16S rRNA gene and Sanger sequencing at BaseClear BV. We then compared the retrieved 16S rRNA sequence to the ASVs of lactic acid bacteria obtained from amplicon sequencing. From those, we selected one *Lactiplantibacillus plantarum* and one *Limosilactobacillus fermentum* strain, isolated from control larvae, and one *Weissella confusa* strain, isolated from HS larvae, for further use.

We characterized the ability of the strains to grow in media with sucrose as a carbon source. For that, we used an inoculum (OD_600nm_ = 0.1) to set up liquid cultures in MRS broth and in a modified glucose-free MRSS broth with sucrose as carbon source, as proposed elsewhere [32], at a final v/v concentration of 1%. All cultures were incubated anoxically at 32°C for 48 hours. Measurements of the pH and the OD density (OD) at 600 nm were taken every hour for the first 12 hours and then at 24, 36, and 48 hours during the incubation in order to construct growth curves in both media. We set up five replicates for every combination of bacterial strain and medium.

### Testing the effect of diet and selected bacteria on development

To assess the effect of supplementing diets with the selected lactic acid bacterial strains, we designed a feeding assay with 12 dietary treatments (Fig. 1B): (i) the control (CTR), (ii) the control with *L. fermentum* (CTR + LF), (iii) the control with *L. plantarum* (CTR + LP), (iv) the control with *W. confusa* (CTR + WC), (v) the high-fat (HF), (vi) the high-fat with *L. fermentum* (HF + LF), (vii) the high-fat with *L. plantarum* (HF + LP), (viii) the high-fat with *W. confusa* (HF + WC), (ix) the high-sugar (HS), (x) the high-sugar with *L. fermentum* (HS + LF), (xi) the high-sugar with *L. plantarum* (HS + LP), and (xii) the high sugar with *W. confusa* (HS + WC). Substrates were supplemented with 1 mg of bacterial cells per 1 L of substrate. The cells were obtained by harvesting liquid cultures after 48 hours and after five washing cycles with PBS-EDTA solution.

To quantify the effect on larval growth, we set up five replicates for each of the 12 treatments. Each replicate consisted of 250 newly hatched larvae in 50 g of substrate. All groups were kept at 25°C and 60% relative humidity with a 12-hour light/12-hour dark cycle. A sample of 10 larvae from every replicate was collected and the wet weight of larvae was measured after 1, 2, and 6 days. Finally, the total number of pupae and adults resulting from every group was counted. After all measurements, five samples were acquired from each replicate (60 samples, in total) and were stored at -20°C to be further processed for 16S rRNA gene amplicon sequencing.

### Bioinformatics and statistical analysis

The sequencing data were processed with the QIIME2 (2020.11) [33] pipeline. The forward and reverse reads were denoised with the DADA2 plugin using the default parameters [34]. After picking representative sequences, they were classified with the *classify-sklearn* method from the *featureclassifie*r plugin. Amplicon sequence variants (ASVs) were aligned using MAFFT [35] and a phylogenetic tree was constructed with Fasttree [36]. Taxonomic assignment of the 300 bp long 16S rRNA gene sequences was conducted with the Silva138.1ref99 database [37]. Community and taxonomy tables were imported into Rstudio 4.1.2 [38] for further analysis. All graphs were made with *ggplot2* [39].

Τhe alpha diversity of the bacterial communities was estimated with *phyloseq* [40]. Statistical comparisons of the alpha diversity between groups (Shannon index) were done with ANOVA tests. Paired comparisons between groups were done with t-tests and paired larval and substrate alpha diversity for samples taken simultaneously from the same experimental group were associated with Pearson correlations using the *stats* package [38]. Beta diversity of the bacterial communities was estimated with Bray-Curtis distances and visualized with Non-Metric Multi-Dimensional Scaling (NMDS) ordinations with *phyloseq* after normalization [40]. Permutational analysis of variance (PERMANOVA) was used to investigate the effect of the diet on observed differences, with *R^2^* representing the explained variance, after checking that all compared groups had homogeneous beta-dispersion with the function *adonis2* of the *vegan* package [41]. Principal Response Curves were used to quantify the treatment response of individual bacterial phylotypes across generations also with the package *vegan* [42]*,* using a redundancy model to explain the microbiota composition with the combination of diet and generation taking into account the repeated measures at each generation (rda model: microbiota ~ diet*generation+ Condition(generation)). The association between bacterial taxa and experimental diets was investigated with the package *indicspecies*, using permutation tests and bootstrapping to obtain confidence intervals [42]. To further investigate the association between specific bacterial taxa and diet, we performed Principal Components Analysis (PCA) with the package *microViz* [43]. Shared ASVs between diet groups were detected with the package *microbiome* [44]. Finally, *PICRUSt2* [45] was used to predict metagenome functions of the bacterial communities by calculating *MetaCyc* [46] pathway abundances through structured mappings of EC gene families. Abundance comparisons were done with t-tests [38].

Mixed effects models were used to describe the repetitive measures of the microbiota composition. Diet and generation were set as fixed factors for the relative abundance of bacterial genera with the *nlme* package [47] and the restricted maximum likelihood method. To assess if the housefly experimental line had an effect on the occurrence of the genera, we constructed and compared models with and without the experimental line as a random factor. In the first case, samples taken from the same experimental line at different generations were auto correlated in the model and each line was treated as a random variable. The *nagelkerke* function of the *rcompanion* package [48] was used to calculate a *P* and a pseudo *R*^2^ value for the models. Finally, the *emmeans* package [49] was used to perform post-hoc tests and to identify the diet effects with marginal models.

Growth curves of the bacterial strains were compared with permutational pairwise tests and curve fitting using the *growthcurver* package [50]. The larval biomass measurements, pupation, and adult emergence counts were tested for distribution using the package *goft* [51] and they were subsequently compared with t-tests with the native *stats* package [38]. Finally, a mixed effects model to describe the repeated measures of larval biomass was performed, using the *nlme* package [47]. Samples that were taken from the same replicate at different time points were auto-correlated and the replicate number was treated as a random variable. The sampling time and the diet were treated as fixed variables. The *nagelkerke* function of the *rcompanion* package [48] was used to calculate a *P* value for the model and the *emmeans* package [49] was used for post-hoc tests. Finally, the larval biomass measurements, pupation, and adult emergence counts were correlated with the Pearson coefficient to the occurrence of specific bacterial taxa with the package *GGally* [52].

## Results

### Dietary sugar is associated with less diverse microbiota

**Figure 2:**
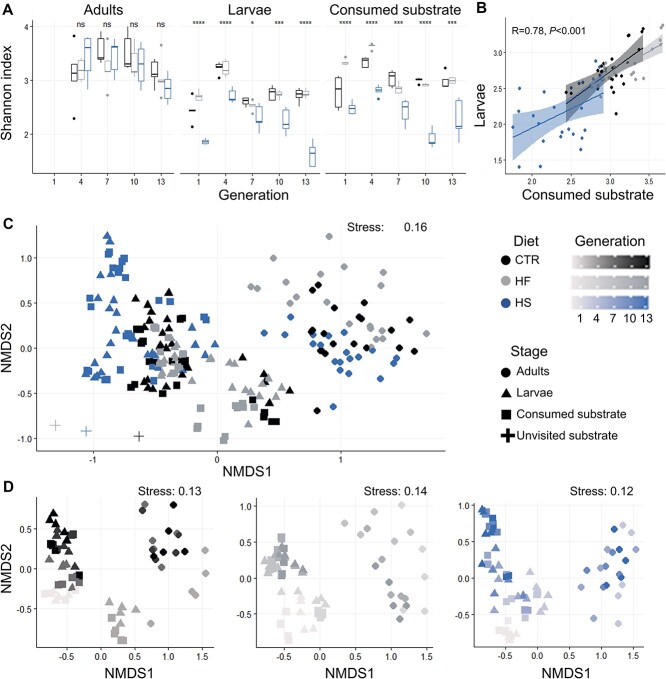
(A) Bacterial Shannon index diversity from the housefly adult and larval guts and the consumed larval substrate after housefly rearing on three experimental diets for 13 generations. Colors represent the different diets. The horizontal line in each boxplot represents the median value of five replicates; the upper limit of the box is the first quartile, and the lower limit is the third quartile. Asterisks indicate significant differences between diets after ANOVA tests within each generation (*P* < 0.05^*^, *P* < 0.01^*^^*^, *P* < 0.001^*^^*^^*^^*^). (B) Shannon index correlation for the bacterial communities of larval guts and consumed larval substrate. (C) Bacterial community beta diversity of the housefly adult and larval guts and all substrate samples visualized with NMDS ordination based on bray-Curtis distance matrices. Shapes stands for the sample type and colors represent the different diets. (D) Bacterial community beta diversity of the housefly adult and larval guts and all substrate samples visualized with three NMDS ordinations based on bray-Curtis distance matrices; one for each experimental diet. Color scale shows the housefly generation the samples come from.

After sequencing of 213 housefly and substrate samples from three diet regimes and 13 generations, 5 029 681 reads and 910 ASVs were retrieved after quality filtering (read distribution and rarefaction curves: Fig. S1; Additional File 1). Shannon index was not different in adults of different diets (Fig. 2A). For the larvae, however, that fed directly from the different substrates, alpha diversity was consistently lower in the HS diet (ANOVA results based on Shannon index from different diets for generation 1: *F =* 49, *P* < 0.001***, generation 4: *F =* 26.3, *P* < 0.001***, generation 7: *F =* 4.6, *P* < 0.05*, generation 10: *F =* 17.5, *P* < 0.001***, generation 13: *F =* 83.8, *P <* 0.001***) (Fig. 2A). The same was true for the consumed substrate (ANOVA results for the Shannon index for generation 1: *F =* 26.4, *P* < 0.001***, generation 4: *F =* 123, *P* < 0.001***, generation 7: *F =* 17, *P* < 0.001***, generation 10: *F =* 184.2, *P <* 0.01***, generation 13: *F =* 16.2, *P* < 0.001***) (Fig. 2A). Also, there was no significant correlation between the alpha bacterial diversity of the adults and the larvae or the consumed substrate, but there was between the larval and the consumed substrate samples that were sampled from the same experimental group (Pearson *r* = 0.78, *P* < 0.001***, for Shannon index; Fig. 2B).

The bacterial community composition between the consumed substrate and the larval guts were less distinct (*R*^2^ = 0.03, *P* < 0.001) than the bacterial composition between the gut samples from larvae and adults (*R*^2^ = 0.32, *P* < 0.001) (Fig. 2C). Diet was a better explanatory factor for the community structure of the larvae (*R*^2^ = 0.23, *P <* 0.001) and the consumed substrate (*R*^2^ = 0.20, *P <* 0.001) than for the adults (*R*^2^ = 0.10, *P* < 0.001) for all samples combined, regardless of generation. Pairwise comparisons of samples showed that diet was more efficient at explaining microbiota differences between HS and HF larvae (HS vs HF: *R*^2^ = 0.27, *P <* 0.001), then between HS and CTR larvae (HS vs CTR: *R*^2^ = 0.15, *P* < 0.001), and lastly between CTR and HF larvae*<* (CTR vs HF: *R*^2^ = 0.11, *P* < 0.01). Samples from different generations were more distinct for the larvae (*R*^2^ = 0.81; *R*^2^ = 0.69; *R*^2^ = 0.70 and *P* < 0.001, for CTR, HF, and HS larvae, respectively) and the consumed substrate (*R*^2^ = 0.80; *R*^2^ = 0.78; *R*^2^ = 0.72 and *P <* 0.001, for CTR, HF and HS consumed substrate, respectively) than for the adult guts (*R*^2^ = 0.26, *P <* 0*.*001; *R*^2^ = 0.33, *P <* 0.001; *R*^2^ = 0.27, *P <* 0.01, for CTR, HF, and HS larvae, respectively) (Fig. 2D).

### Diet-associated bacterial taxa

**Figure 3:**
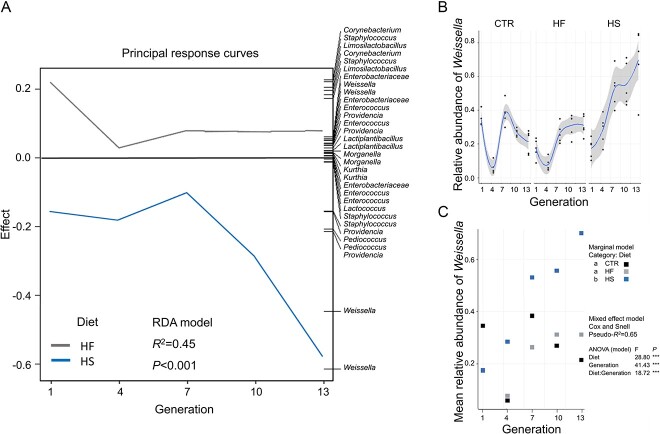
Gut microbiota shifts for housefly larvae over the course of 13 generations for larvae reared in three types of substrate. Colors represent the different diets: CTR stands for the control, HF stands for the high-fat, and HS stands for the high-sugar larval substrate. In principal response curve analysis (A), the taxa names on the right refer to respective bacterial phylotypes that explain the variation of the HF and HS diets in comparison with the CTR diet. Two ASVs assigned to *Weissell*a responded to the HS diet. When looking into the relative abundance of this genus (B), the HS diet led to a higher relative abundance of *Weissella* over the generations. The mixed effect model based on the relative abundance of *Weissella* (C) revealed that both diet and generation predict the occurrence of the genus in the larval bacterial composition, with the HS diet being associated with higher relative abundance.

Sequencing of the three types of unconsumed substrate revealed that over 95% of their overall bacterial community composition was attributed to taxa shared between all three. The 10 most abundant taxa; namely *Pantoea*, *Geobacillus*, *Limosilactobacillus*, *Pediococcus*, *Pseudomonas*, *Weissella*, *Enterobacteriaceae*, *Enterococcus*, *Providencia*, and *Corynebacterium* constituted 90%, 97%, and 95% of the CTR, HF, and HS substrate, respectively (Fig. S2; Additional File 1). Several ASVs of the fresh substrate assigned to *Weissella*, *Pediococcus*, and *Enterobacteriaceae* were also present in all samples of the consumed substrate (Fig. S2; Additional File 1) and larval guts (Fig. S3; Additional File 1). From the aforementioned taxa, *Weissella* became gradually more abundant in the HS larvae and several ASVs assigned to this genus drove the variance of the HS larvae compared to the CTR larvae over the generations tested (Fig. 3). In contrast, the housefly larvae microbiota of the HF diet deviated less from the CTR diet, and the distance between them stabilized after the 7^th^ generation (Fig. 3A).

Analysis of indicative bacterial taxa for each diet revealed distinct phylotypes between treatments. The CTR diet was associated with fourteen ASVs assigned to *Lacticaseibacillus*, *Latilactobacillus*, *Enterococcus*, *Limosilactobacillus*, *Lactiplantibacillus*, *Enterobacteriaceae*, *Pediococcus*, and *Kocuria*. The HF diet was associated with 12 ASVs assigned to *Staphylococcus*, *Corynebacterium*, *Enterococcus,* and *Acinetobacter*. The HS diet was associated with nine ASVs assigned to *Weissella*, *Pediococcus*, *Brevibacterium*, and to the family *Micrococcaceae*. The full list of indicative taxa along with the respective computed *IndVal* statistic and *P* values is shown in Table S1 (Additional File 2).

Mixed effect models showed that the relative abundance of the genus *Weissela* was significantly affected both by generation and diet, with the HS diet leading to different occurrence levels in comparison to the CTR and the HF diet (Fig. 3C). Furthermore, the bacterial genera *Morganella*, *Myroides*, *Pantoea*, and *Lactococcus* were not affected by the diet. Including the housefly experimental line as causing random effect variance had no influence on all constructed models (Table S2, Additional File 3).

### Utilization of sucrose benefits specific bacteria

**Figure 4:**
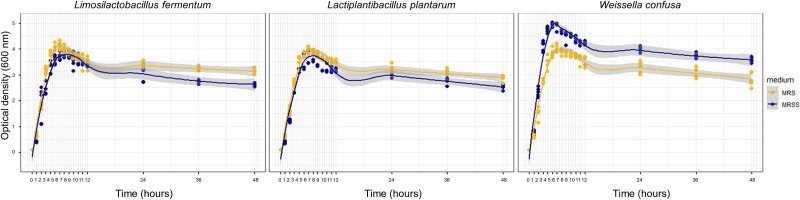
Growth of three lactic acid bacteria strains isolated from housefly larvae for 48 hours in a selective medium with glucose (MRS) and sucrose (MRSS) as carbon sources.

We isolated several lactic acid bacterial colonies and selected three species: *Limosilactobacillus fermentum*, *W. confusa,* and *Lactiplantbacillus plantarum*. The genera were highlighted both in the Principal Response Curve analysis as driving the variance between treatments (Fig. 3A) and were found to be indicative of specific treatments (*Weissella* for HS, *Limosilactobacillus* and *Lactiplantibacillus* for CTR), as summarized in the previous section. The full 16S rRNA gene sequences of all three strains were traced back to multiple ASVs retrieved from the amplicon sequencing dataset (Additional File 4). The dominance of ASVs associated with *Weissella* in the HS diet suggested that this genus had a higher fitness when sucrose was present. To test this hypothesis, we grew all three selected lactic acid bacterial strains in liquid medium with glucose (MRS) or sucrose (MRSS) as sole carbon source. Although all species revealed similar growth rates when growing in the presence of glucose, we observed that *W. confusa* grew significantly faster in the modified broth with sucrose (*P <* 0.01, in comparison with both *L. fermentum* and *L. plantarum* strains) (Fig. 4).

### Probiotic bacteria revert the negative effect of sucrose

Housefly larvae that were reared on the HS diet had lower biomass compared to CTR (n = 5, t-value = 2.50, *P* < 0.05) and HF larvae (n = 5, t-value = 5.44, *P* < 0.001), 6 days after hatching. On the contrary, larval biomass did not differ between CTR and HF. Given the increase of *Weissella* in the HS diet and the decrease of other lactic acid bacteria (Fig. 3), we tested whether the negative effect of the diet was caused by *Weissella* and whether the addition of *L. plantarum* and *L. fermentum* would reverse this effect.

**Figure 5:**
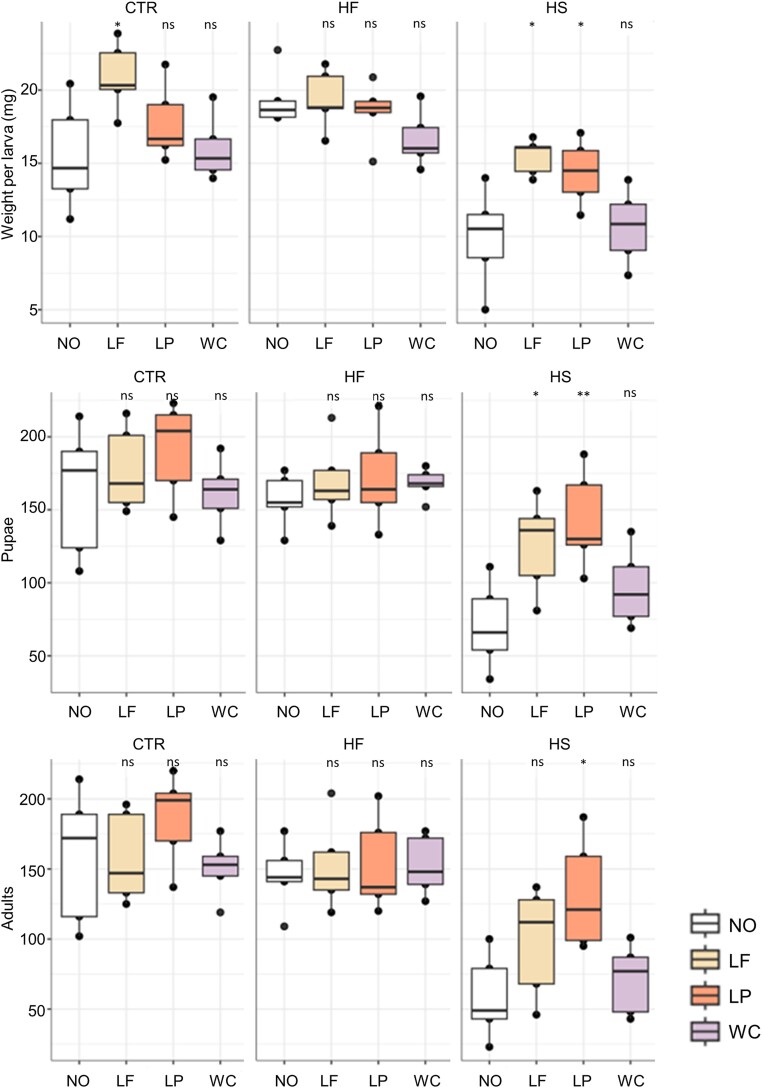
Measurements of housefly larval biomass for 6-day-old larvae, pupation, and adult emergence. The line in each box represents the median value of five biological replicates; the upper limit of the box is the first quartile, and the lower limit is the third quartile. CTR stands for the control, HF stands for the high-fat, and HS stands for the high-sugar larval substrate. Colors represent the different added bacteria. NO stands for no additive, LF stands for added *Limosilactobacillus fermentum*, LP stands for added *Lactiplantibacillus plantarum*, and WC stands for added *Weissella confusa* in the respective substrates. Asterisks indicate statistically significant pairwise differences according to paired t-tests with reference to the substrate with no additives (NO) (*P* < 0.05^*^, *P* < 0.01^*^^*^). Each experimental group consisted of 250 larvae.

The addition of *L. fermentum* to the HS substrate was associated with higher larval biomass than that of HS larvae (n = 5, t-value = 3.45, *P <* 0.05, Fig. 5A). Moreover, after the addition of *L. fermentum* in the HS substrate, the 6-day-old larvae were not significantly different from CTR and HF; they were, however, still smaller (n = 5, t-value = −4.55, *P <* 0.01) than those in the CTR substrate inoculated with *L. fermentum* (Fig. 5A). Similarly, adding *L. plantarum* to the HS substrate led to biomass similar to the CTR and HF larvae. The addition of *W. confusa* in all three diets had no effect (Fig. 5A).

The model for the larval biomass repeated measures showed a strong effect of the treatment (*P <* 0.001, model’s pseudo-*R*^2^ = 0.85) and grouped all treatments into three levels: the HS substrate and the HS substrate with added *W. confusa* were together (group a; lsmeans = 5.28 mg); the HF substrate, the HF substrates with *L. fermentum* and *L. plantarum*, and the CTR substrate with *L. fermentum* were together (group b; lsmeans = 8.73); and the remaining treatments were grouped together at an intermediate level (group ab; lsmeans = 7.51).

Differences in pupation followed a similar pattern. Fewer larvae managed to pupate in the HS substrate compared to CTR (n = 5, t-value = 3.80, *P* < 0.01) and HF (n = 5, t-value = 5.43, *P* < 0.01). However, *L. fermentum* and *L. plantarum* in the HS substrate raised pupation at levels similar to CTR and HF (Fig. 5B), whereas adding *W. confusa* to the HS substrate did not lead to any changes. The same was true for the other two diets: adding *W. confusa* did not alter pupation. Housefly adult emergence was also lower for the HS diet, and adding *L. fermentum* and *L. plantarum* had similar effects as those described for pupation (Fig. 5C).

### Impact of probiotics on larvae

**Figure 6:**
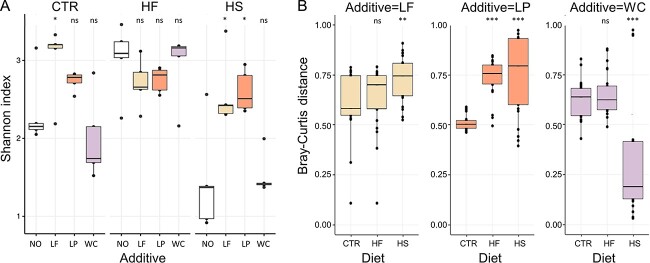
Bacterial diversity from the housefly larval gut from larvae reared on three diets with and without probiotics. CTR stands for the control, HF stands for the high-fat, and HS stands for the high-sugar larval substrate. NO stands for no additive, LF stands for added *Limosilactobacillus fermentum*, LP stands for added *Lactiplantibacillus plantarum* and WC stands for added *Weissella confusa*. (A) Shannon index is presented as alpha diversity index. The horizontal line in each boxplot represents the median value of five replicates; the upper limit of the box is the first quartile, and the lower limit is the third quartile. Asterisks indicate statistically significant pairwise differences according to paired t-tests with reference to the substrate with no additives (NO) (*P* < 0.05^*^). (B) Beta diversity of the bacterial communities from larval guts is represented by bray-Curtis distances, showing the difference of larvae reared with probiotics, in reference to larvae reared without probiotics for each of the three experimental diets. The horizontal line in each boxplot represents the median value of 25 pairwise comparisons; the upper limit of the box is the first quartile, and the lower limit is the third quartile. Asterisks indicate statistically significant pairwise differences according to paired t-tests with reference to the CTR substrate (*P* < 0.05^*^, *P* < 0.01^*^^*^, *P* < 0.001^*^^*^^*^).

The sequencing of the 60 larvae from the probiotic supplementation feeding assay returned 2 862 132 reads and 1868 unique ASVs. The Shannon alpha bacterial diversity of HS larvae was lower than that of the CTR and HF larvae (Fig. 6A), similarly to our previous results (Fig. 2A). However, the addition of *L. fermentum* and *L. plantarum* in the CTR and HS substrate resulted in Shannon index increase (Fig. 6A). Subsequently, the addition of *L. fermentum* and *L. plantarum* also resulted in lower *Weissella* abundance (Fig. S4, Additional File 1) in CTR and HS larvae. Both the substrate and the added bacterial strain (*R*^2^ = 0.15 and *R*^2^ = 0.23, *P* < 0.001***) significantly affected bacterial microbiome composition. The combination of the two factors also had a significant effect (*R*^2^ = 0.22, *P* < 0.001***). Adding *L. fermentum* and *L. plantarum* to the substrates led to a higher relative abundance of *Limosilactobacillus* and *Lactiplantibacillus* in the HS larvae and the CTR larvae, respectively. This was not observed with *W. confusa* and *Weissella* (Fig. S4, Additional File 1). Additionally, after adding *L. fermentum* and *L. plantarum* in the HS substrate larvae, the gut microbiota showed higher levels of *Corynebacterium* and adding *L. fermentum* also led to higher levels of *Lactococcus* and *Proteus* (Fig. S4, Additional File 1).

The genera *Lactiplantibacillus* and *Limosilactobacillus* were associated with the CTR and HF larvae, but also with LP and LF enhanced substrates, respectively (Fig. S5, Additional File 1). Their relative abundance was positively correlated with measured larval biomass, but only for the HS larvae (Fig. S6, Additional File 1). Adding *L. fermentum* and *L. plantarum* had a stronger impact on the gut bacterial communities of HS larvae than on CTR larvae (Fig. 6B).

Predicted metagenome functions showed that two metabolic pathways connected to sucrose degradation (invertase and phosphorylase enzymatic activity) were lowered in the HS larvae when the substrate was enhanced with *L. fermentum* and *L. plantarum* (n = 5, t-value = 6.05, *P* < 0.001 and t-value = 6.38, *P* < 0.001, respectively, for invertase; n = 5, t-value = 3.08, *P* < 0.001 and t-value = 2.86, *P* < 0.05, respectively, for phosphorylase) (Additional File 1, Fig. S7). In accordance to the effect of *W. confusa* on the bacterial communities, adding *W. confusa* in all substrates was not linked to any changes of these pathways (Additional File 1, Fig. S7).

## Discussion

Insect gut bacteria can be acquired through environmental routes and, most prominently, from ingested food. Nonetheless, the insect gut microbiota can radically differ from the food source. Mechanisms that insects possess can select microbes, other microbes may possess advanced transmission capacities, and insect immunity can show selective tolerance [1]. Indeed, although insects interact with microorganisms in a variety of ways, in line with their enormous ecological and physiological diversity, often they associate with a limited set of microbial taxa [53]. Here, we investigated the effect of high fat and high sugar diets on the natural gut microbiota of the common housefly. By studying the response of the species’ gut bacterial community to these dietary changes over the course of 13 generations, we aimed at tracing intergenerational community changes and the impact on host fitness.

Whereas adult gut microbiota showed no change in alpha diversity and little change in beta diversity, larvae, which directly fed from the different substrates, harbored more distinct gut communities according to diet. As a holometabolous species, the housefly’s gut line is rebuilt during metamorphosis, making the maintenance of microbes acquired at the larval stage challenging [54]. Even if adult flies can re-harbor bacteria from the meconium during emergence, this explains why adult gut bacterial communities were not severely affected. However, in some cases the effect of diet on larval guts was gradual through generations, implying that changes might be sustained throughout the life cycle. More specifically, excess sucrose in the rearing substrate gradually led to a less diverse bacterial community.

Microbial shift as response to environmental change has the potential of extending host adaptability and evolution [30]. In the present study, that would mean that diet would affect the composition of the gut bacterial community towards selecting for adapted microbiota that can counteract any negative effects of the diet on the host. On the contrary, we showed that the high sucrose diet led to the loss of beneficial lactic acid bacteria and low developmental success. This suggests that, within the confines of laboratory-reared flies, the variation in the microbiota did not confer adaptive advantage under this stressful diet.

In general, it is known that gut microbiotas can be altered due to over-consumption of sucrose and other sweeteners through transcriptional, genetic, and ecological adaptations [55]. A study with *Drosophila* flies showed that a diet enriched with saturated fats, sugars, and salt could harm the overall lifespan, locomotor activity, sleep, and reproductive success [56]. Also, sucrose has been shown to cause a less diverse gut microbiota in adult houseflies [27]. Both in our initial intergenerational bacterial survey and in our subsequent feeding assay, the HS diet led to a poor bacterial community in the host. By tracing specific bacteria that drove changes between the experimental diet groups, we could detect phylotypes that dominated the HS larvae, taxonomically assigned to the genus *Weissella*.

The *Weissella* genus has numerous strains isolated from fermented food [57]. In our case, we detected it in all substrate samples and in larvae of all diets. It has been suggested that grain mixtures containing high levels of hemicellulose are proper substrates for fermentation by *W. confusa* [32]. Nevertheless, the use of *Weissella* spp. as commercial starters for food fermentation has not been established yet, as there are certain processes and attributes (e.g. biogenic amine production) that need to be investigated [58].

Upon comparing a *W. confusa* strain to a *L. fermentum* and a *L. plantarum* strain, all isolated from housefly larvae, we found that the former was faster in utilizing sucrose as a carbon source; a finding agreeing with the dominance of *Weissella* in the HS larvae and the respective consumed substrate. It has been reported that many *Weissella* strains can produce high levels of exopolysaccharides from sucrose, particularly dextran, showing the genus’ efficiency in utilizing sucrose [59–64]. In a study including a total of 156 lactic acid bacteria strains isolated from children’s fecal samples, the highest exopolysaccharide-producing one was *W. confusa* VP30, when utilizing sucrose [65]. Another study, including *W. confusa* and *L. fermentum* strains, showed that only some *Weissella* strains could produce exopolysaccharides from sucrose, with the strain *W. confusa* UC4052 being the most efficient [66].

Combined with the results that excess sucrose had a negative effect on housefly development, we aimed to test if the high relative abundances of *Weissella* were associated with this. Therefore, we tested the effect of *W. confusa* on the larval development and the effect of the two other aforementioned lactic acid bacterial strains that were lacking from the HS larvae and were less efficient in sucrose utilization: *L fermentum* and *L. plantarum*. We found that the *W. confusa* addition in the rearing substrate had no negative effect on larval development. The levels of *Weissella* in 13th generation HS larvae of the initial diet experiment are comparable to the ones found in the HS larvae of the probiotic supplementation experiment, where we included larvae which had been reared on HS diet for more than 13 generations. Therefore, we do not have evidence to support that the adaptation of the microbiome to the HS diet continued to favor *Weissella*.

Reintroducing the *L. fermentum* and *L. plantarum* strains in the HS-rearing substrate, however, resulted in higher larval biomass, as well as higher pupation and adult emergence. Both species, formerly assigned to the genus *Lactobacillus*, were shown to revert the negative effect of the HS diet to a large extent. Moreover, their introduction restored the bacterial alpha diversity in the larval gut and subsequently led to lower levels of *Weissella*. The lower levels of *Weissella* were accompanied by lower abundances of the metabolic pathways of sucrose degradation (sucrose invertase and sucrose phosphorylase enzymatic activities) in HS larvae; implying that the restored bacterial taxa do not contribute to sucrose degradation like *Weissella*. Adding *W. confusa* in the substrate did not lead to any change in the alpha diversity and the predicted functions of the bacterial metagenome. The alpha-diversity increase connected to *L. fermentum* and *L. plantarum* leads us to attribute any observed beneficial effect in the larvae not simply to their occurrence in the larval gut but rather to their impact on the microbiome community diversity, that came as a result after their inoculation. The latter is especially important when taking into account that even though the abundance of these two probiotic genera was found to be higher after the inoculation in some substrate types, their overall relative abundance continued to be low compared to more prevalent genera. Also, not only their relative abundance continued to be lower, but other genera, like *Corynebacterium* or *Proteus*, showed higher abundance increase after the probiotic supplementation.

The bacterial alpha diversity increase after substrate enhancement with *L. fermentum* and *L. plantarum* corroborates experiments in vertebrates. Strains of those bacterial species could increase the gut microbiota diversity and favor beneficial microorganisms in mouse models [67, 68]. Such findings focus on the impact these probiotics bring on the whole gut microbiota and not solely to their possible direct effects on the host. Bacteria such as *L. fermentum* and *L. plantarum* can have complex nutritional requirements [69], deriving energy by metabolizing disaccharides or higher saccharides [70], and they are, therefore, widely used in fermentation [71].

Apart from their impact on the overall microbiota structure, *L. fermentum* is widely considered a promising probiotic [72]. It has been tested in vertebrates, where an *L. fermentum* strain acted against diet-induced obesity in mice [73], possibly due to its ability to ameliorate glucose and lipid metabolism despite a high-fat diet [74]. In flies, the provision of *L. fermentum* could revert the effect of high-sugar and high-fat diets in *Drosophila*. The high-sugar diet caused elevated expression of insulin-like peptides, whereas the high-fat one caused elevated expression of fatty acid synthase, acetyl-CoA carboxylase and phosphoenolpyruvate carboxykinase. Provision of *L. fermentum* or of ferulic acid produced by it lowered the expression close to the control, possibly by regulating dTOR pathways [75]. In our study, the comparison of CTR and HF larvae showed no difference for growth and developmental success. At the same time, bacterial phylotypes assigned as *Limosilactobacillus* from the fresh substrate were maintained in CTR and in HF larvae. The presence of *L. fermentum* in the housefly larvae gut might thus contribute to negating negative effects of the HF diet, as proven in *Drosophila* flies.

*L. plantarum* is also characterized as a probiotic and is considered to be symbiotic to many insects, including *Drosophila* flies [76]. It has been shown that it can regulate the TOR-dependent nutrient-sensing system in *Drosophila*, thus controlling growth signaling [77]. More specifically, the presence of *L. plantarum* alone was enough to enable larval development, whereas germ-free flies could not survive the same condition. The species could directly affect the levels of *Drosophila* circulating insulin-like peptides [77] and thus main nutrient-sensing growth-regulatory mechanisms [78, 79].

A diet that does not favor beneficial bacteria, such as the ones found in the present study, would prevent their harboring by the host. Our experiment showed that even though the initial bacterial communities of the three unvisited rearing substrates did not differ and although all housefly experimental lines derived from the same population, the different substrate types led to bacterial microbiota divergence in the host gut. Previous experiments in our lab including the same housefly strain had shown that the microbial community of the substrate was directly linked to the simple fungal microbiota in the larval gut, in contrast to the more complex bacterial microbiota [14]. However, the fungal and bacterial microbiotas correlated significantly. Therefore, it would be interesting to further investigate whether the bacterial community divergence would affect the fungal community composition or the latter would be stable and directly linked with the substrate initial composition.

Future studies should also inspect the metabolic and genetic mechanisms through which beneficial microbes, like the ones found in the present study, can aid both in pre-digestion and nutrient uptake, boosting host development. In a broader context, studies on insects could aid in the understanding of the effect that certain bacterial members of the gut microbiota can have on health and development. Also, by associating dietary components with microbiota shifts, we can understand how diet impacts health not just by evaluating nutritional value but also by assessing if it promotes the harboring of a healthy host gut microbiota.

## Conclusions

We investigated the effect of high fat and sugar levels on the natural gut microbiota of a dipteran species, the common housefly. By following the response of the species’ gut bacterial community to these diets over 13 generations, we aimed to trace intergenerational community shifts and link them with insect performance. Our main findings were that although the effects of the fat-rich diet were comparatively limited and stabilized through time, excess sucrose in the larval-rearing substrate gradually led to less diverse bacterial communities and lower developmental success. By tracing specific bacteria that caused the observed changes, we found that two probiotic bacterial strains belonging to *L. fermentum* and *L. plantarum*, which were possibly outcompeted in the high-sugar environment, were lacking from the housefly larvae of the high-sugar diet. Testing their effect on housefly development revealed that their re-introduction in the high-sugar larval substrate could reverse the observed negative effects in insect development and also restore the host microbiome diversity.

## Supplementary Material

AdditionalFile1_wrae193

AdditionalFile2_wrae193

AdditionalFile3_wrae193

AdditionalFile4_wrae193

AdditionalFile5_wrae193

Additional_File_wrae193

## Data Availability

The datasets supporting the conclusions of this article are available in the Sequence Read Archive https://www.ncbi.nlm.nih.gov/bioproject, under BioProject ID PRJNA1096680, and in the accompanying Additional Files.
