# Supplementary material for: High-sugar diet leads to loss of beneficial probiotics in housefly larvae guts
Source: ISME J. 2024 Oct 3;18(1):wrae193. doi: 10.1093/ismejo/wrae193 (PMC11495414; doi:10.1093/ismejo/wrae193)
Supplement: AdditionalFile1_wrae193 [file additionalfile1_wrae193.docx]

# High-sugar diet leads to loss of beneficial probiotics in housefly larvae guts

Anna Voulgari-Kokota*^1,2^, Francesco Boatta^3^, Ruud Rijkers^3,4^, Bregje Wertheim^1^, Leo W. Beukeboom^1^, Jacintha Ellers^2^, Joana Falcao Salles^1^

^1^ Groningen Institute for Evolutionary Life Sciences (GELIFES), University of Groningen, P.O. Box 11103, 9700 CC, Groningen, The Netherlands

^2^ Laboratory of Microbiology, Wageningen University, 6700 EH, Wageningen, The Netherlands

^3^ Amsterdam Institute for Life and Environment, Section Ecology and Evolution, Vrije Universiteit Amsterdam, 1081 HV, Amsterdam, The Netherlands

^4^ Department of Environmental Science, Stockholm University, SE-106 91, Stockholm, Sweden

*corresponding author: [anna.voulgarikokota@wur.nl](mailto:anna.voulgarikokota@wur.nl)

Supporting figures referenced in the main manuscript.

**Figure S1.** Rarefaction curves, showing absolute number of ASVs detected against the total number of reads for every sample.

**
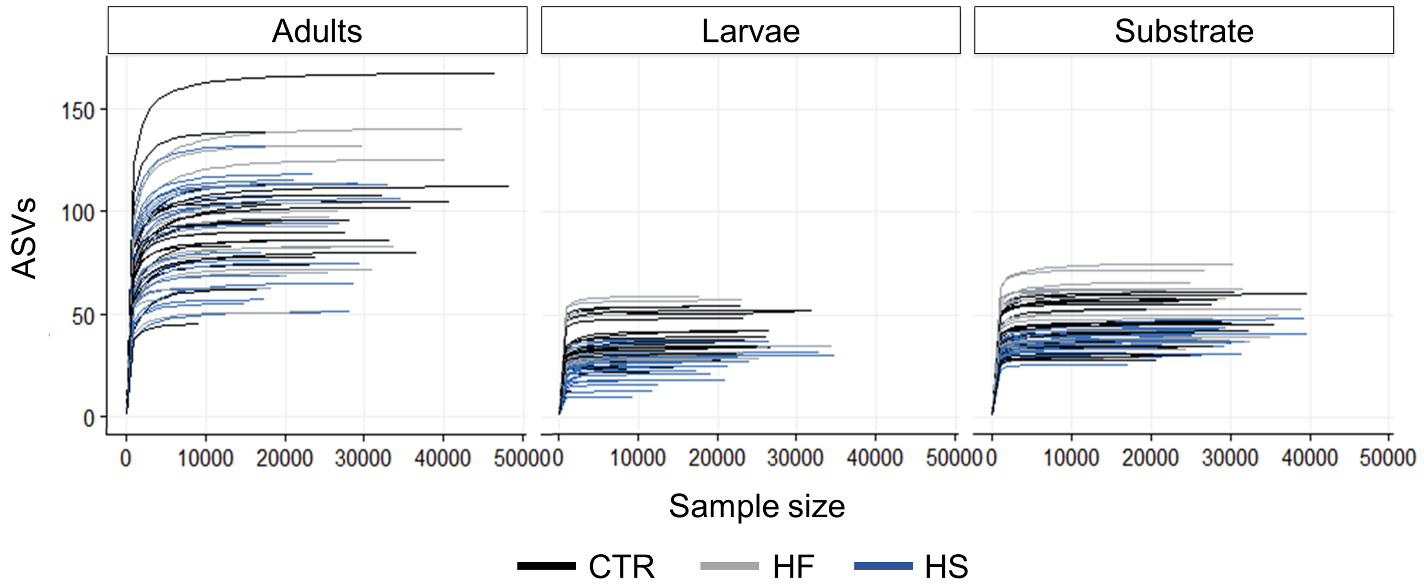
**

Rarefaction curves, showing absolute number of ASVs detected after 16S rRNA sequencing against the total number of reads (Sample size) of all samples from housefly adults, larvae and rearing substrate. Colors represent the three experimental treatments/diets: CTR stands for the control, HF stands for the high-fat and HS for the high-sugar larval substrate.

**Figure S2.** Bacterial community composition of consumed and fresh housefly rearing substrate


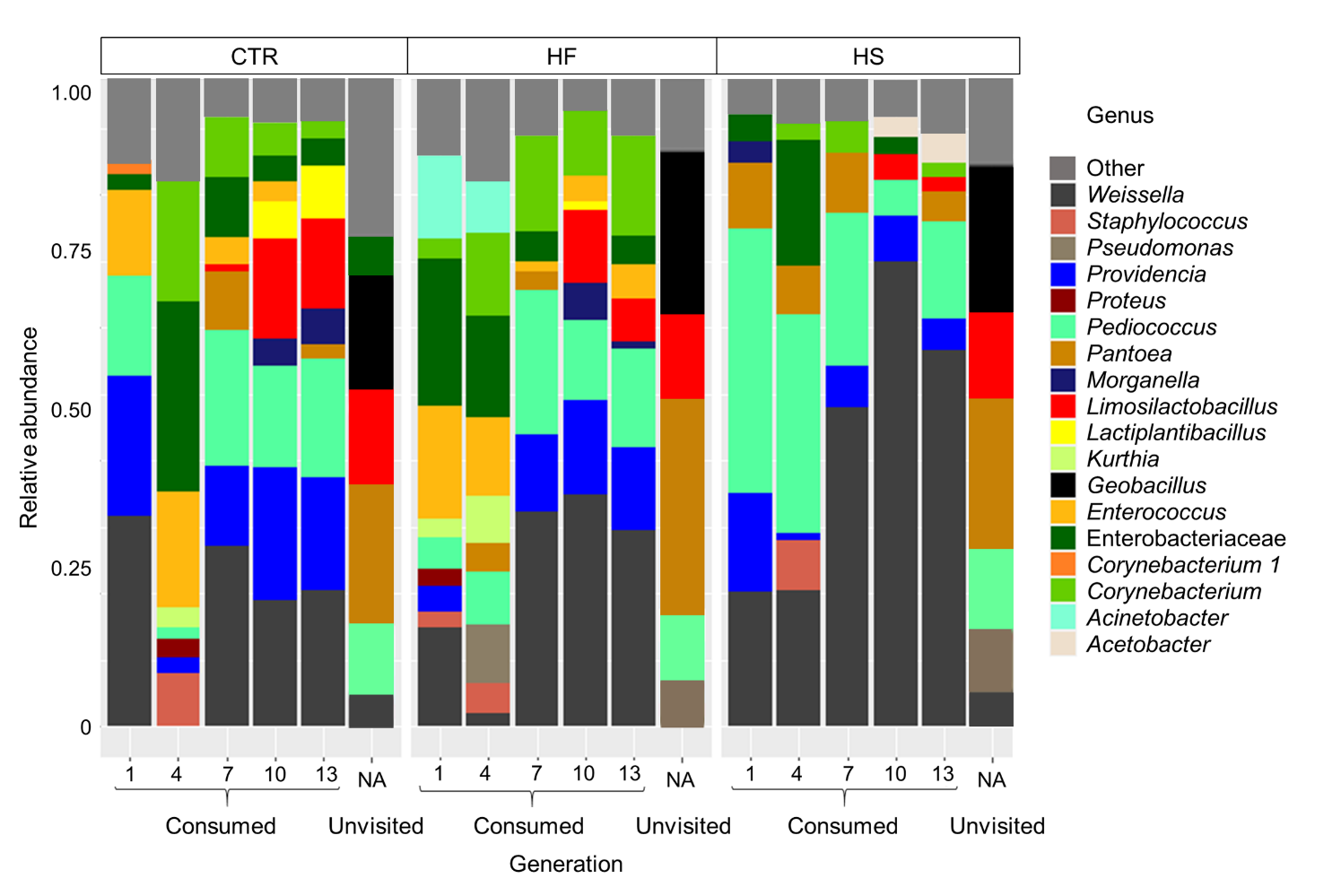


Bacterial community composition of consumed and unvisited housefly rearing substrate for three experimental diets. CTR stands for the control, HF stands for the high-fat and HS stands for the high-sugar larval substrate. Each barplot for the consumed substrate summarizes five biological replicates. The barplot for the fresh substrate describes one sample. Bacterial taxa are included if the summed relative abundance of the phylotypes assigned to them are detected at a percentage of at least 5% in the whole dataset.

**Figure S3.** Bacterial community composition of housefly gut samples


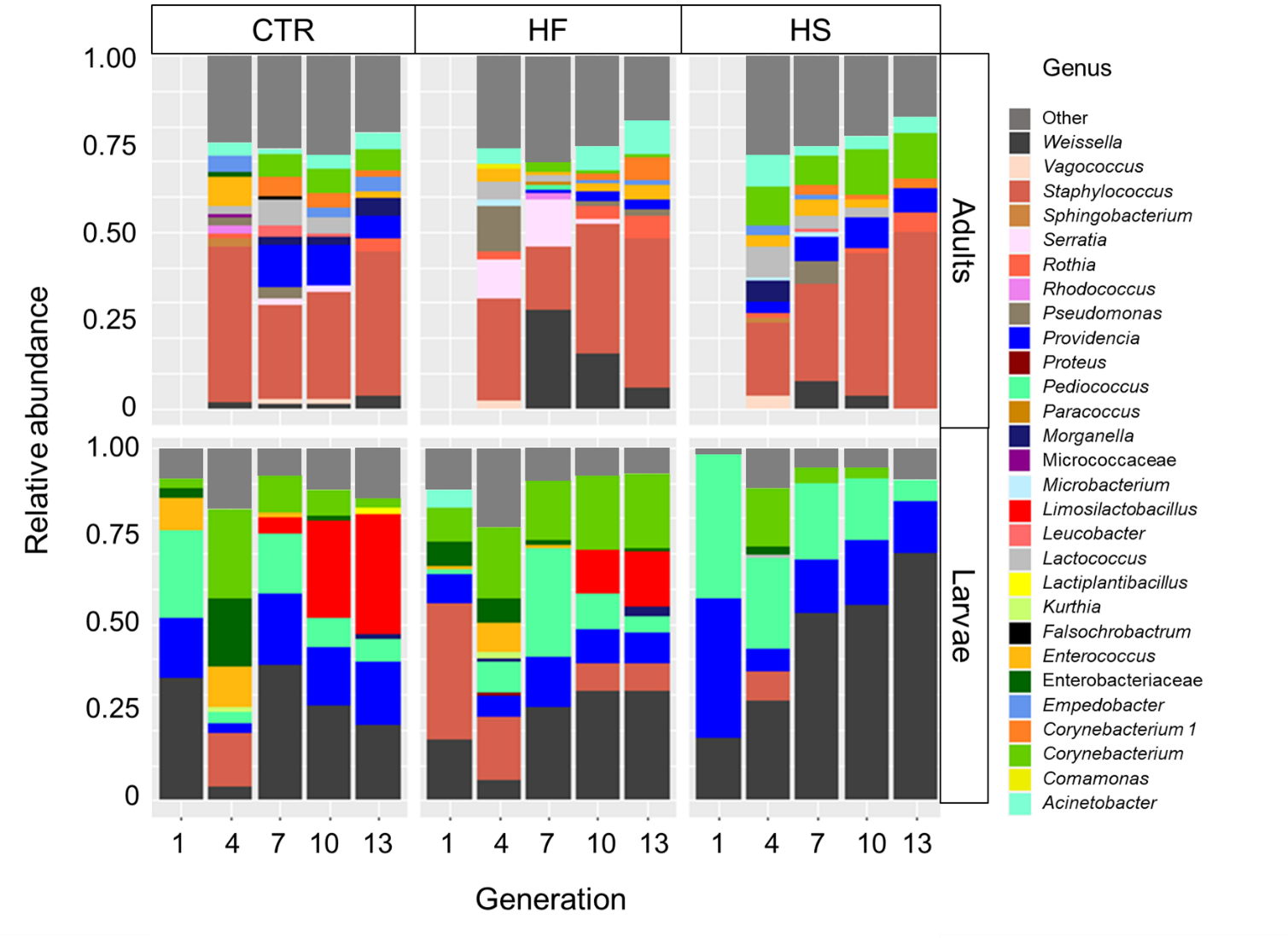


Bacterial community composition of housefly gut samples for two developmental stages (adults/larvae) and three experimental diets. CTR stands for the control, HF stands for the high-fat and HS stands for the high-sugar larval substrate. Each barplot summarizes five biological replicates. Bacterial taxa are included if the summed relative abundance of the phylotypes assigned to them are detected at a percentage of at least 5% in the whole dataset.

**Figure S4.** Relative abundance of the most abundant bacterial genera in the housefly larval guts


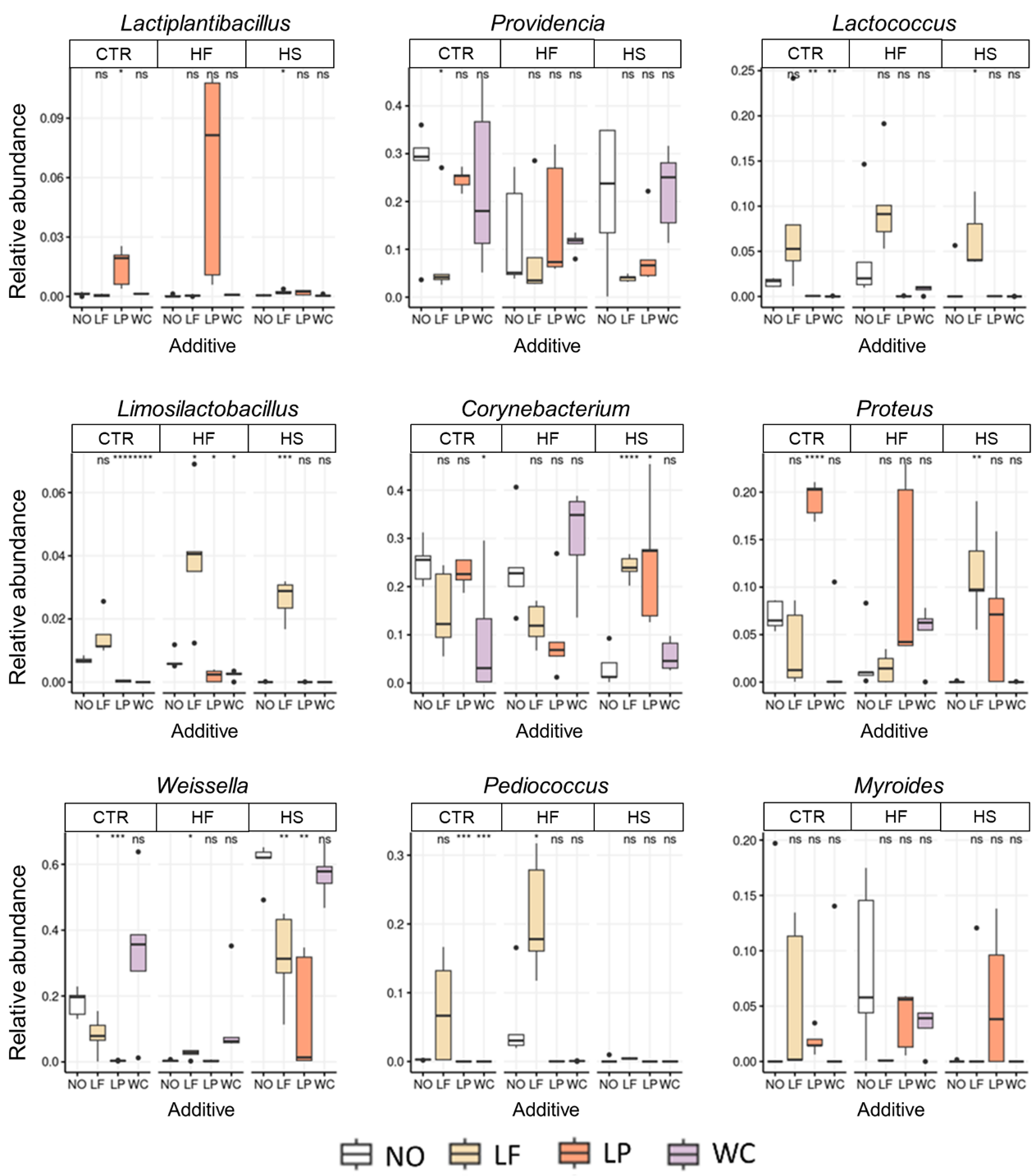


Relative abundance of the most abundant bacterial genera in the community of housefly larval guts, for larvae reared in three types of substrate with and without substrate inoculation with bacteria. CTR stands for the control, HF stands for the high-fat and HS stands for the high-sugar larval substrate. Colors stand for the bacterial strain that was added in the substrate׃ NO stands for no additive, LF stands for *Limosilactobacillus fermentum*, LP stands for *Lactiplantibacillus plantarum* and WC stands for *Weissella confusa*. The horizontal line in each boxplot represents the median value of five replicates, the upper limit of the box is the first quartile and the lower limit is the third quartile. Asterisks indicate statistically significant pairwise differences according to paired t-tests with reference to the substrate with no additives (*P<0.05**, *P<0.01***, *P<0.001****, *P<0.0001*****).

**Figure S5.** PCA ordination plots for the housefly larval gut microbiota.


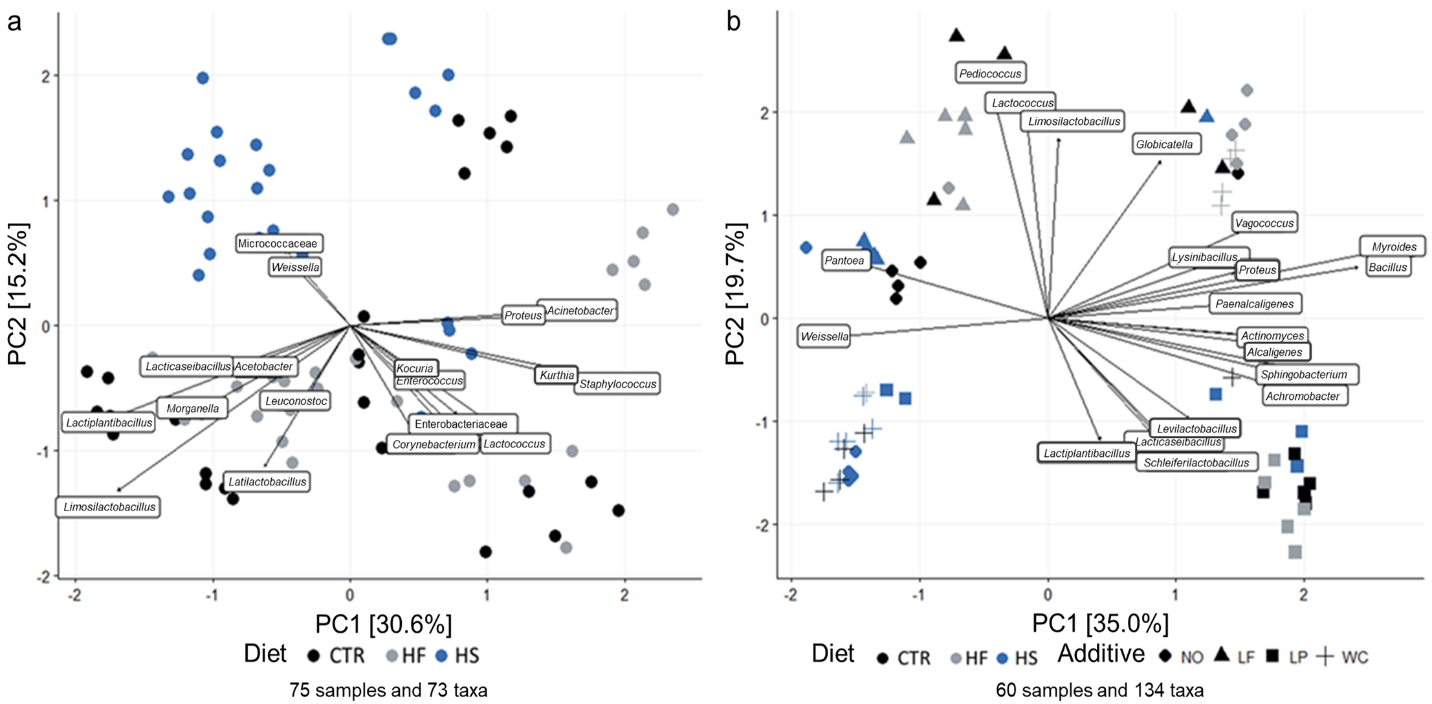


PCA ordination plots for the housefly larval gut microbiota. The taxa names in the labels stand for the bacteria taxa which drive the differences between samples: a) larvae reared on three experimental diets for 13 generations, b) larvae reared on three experimental diets with and without additional bacterial strains in the substrates. Colors stand for the experimental diet: CTR stands for the control, HF stands for the high-fat and HS stands for the high-sugar larval substrate. Shapes stand for the added bacterial strains: NO stands for no additive, LF stands for *Limosilactobacillus fermentum*, LP stands for *Lactiplantibacillus plantarum* and WC stands for *Weissella confusa*.

**Figure S6.** Pairwise correlations of larval development with the frequency of specific bacterial phylotypes


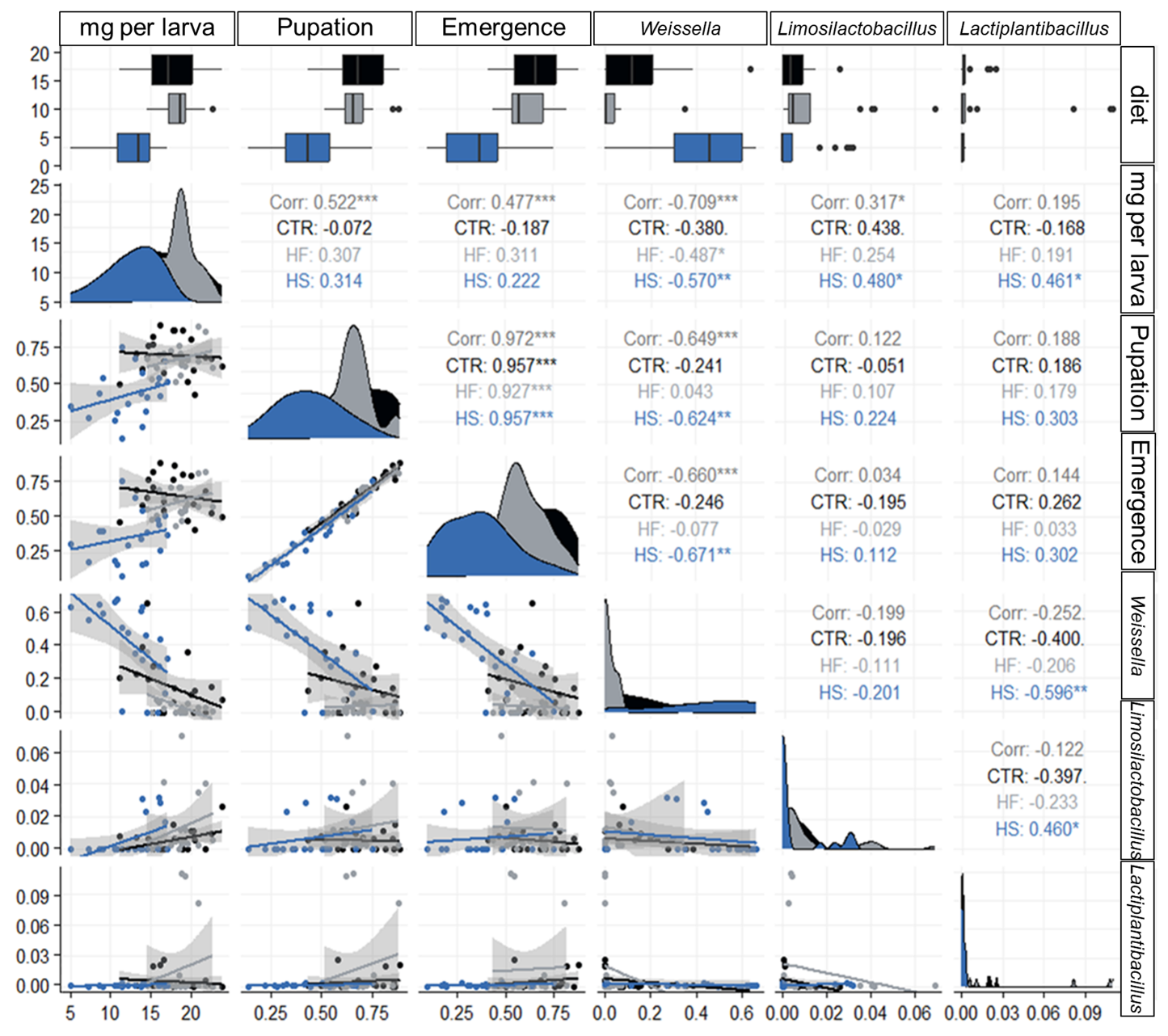


Pairwise correlations of the larval biomass (measured in mg per larva), pupation ratio and adult emergence ratio with the frequency of the phylotypes assigned as *Limosilactobacillus*, *Lactiplantibacillus* or *Weissella* in the respective samples. Asteriscs indicate statistically significant Pearson correlations (*P<0.05**, *P<0.01***, *P<0.001****).

**Figure S7.** Metabolic pathways relevant to sucrose degradation, after functional prediction of the housefly larval bacterial microbiome


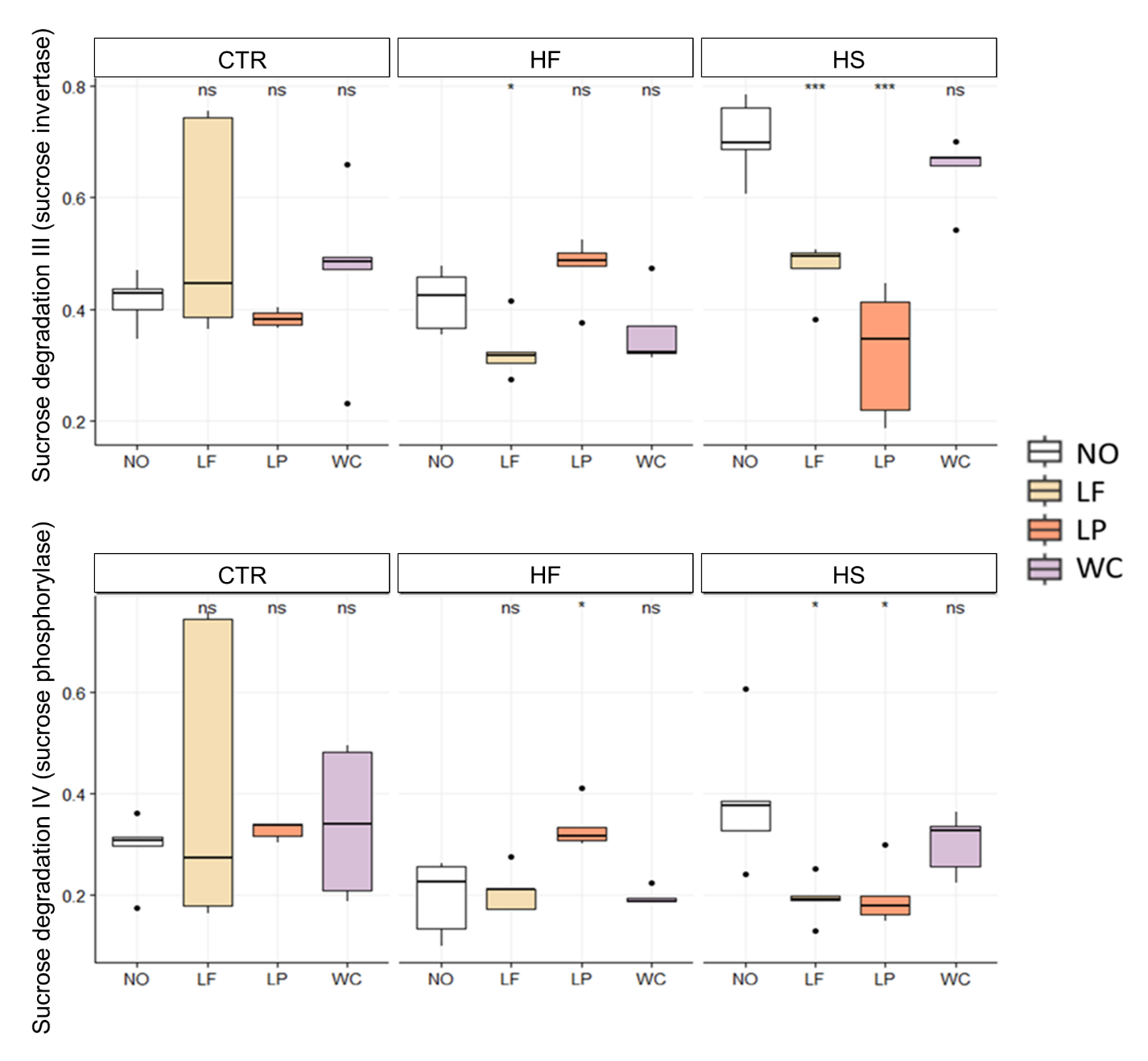


Relative abundance of two bacterial metabolic pathways relevant to sucrose degradation, after functional prediction of the housefly larval bacterial microbiome. The input was normalized ASV sequences. Larvae were reared in three types of substrate with and without substrate inoculation with bacteria. CTR stands for the control, HF stands for the high-fat and HS stands for the high-sugar larval substrate. Colors stand for the bacterial strain that was added in the substrate׃ NO stands for no additive, LF stands for *Limosilactobacillus fermentum*, LP stands for *Lactiplantibacillus plantarum* and WC stands for *Weissella confusa*. The horizontal line in each boxplot represents the median value of five replicates, the upper limit of the box is the first quartile and the lower limit is the third quartile. Asterisks indicate statistically significant pairwise differences according to paired t-tests with reference to the substrate with no additives (*P<0.05**, *P<0.01***, *P<0.001****).
