# Supplementary material for: High-sugar diet leads to loss of beneficial probiotics in housefly larvae guts
Source: ISME J. 2024 Oct 3;18(1):wrae193. doi: 10.1093/ismejo/wrae193 (PMC11495414; doi:10.1093/ismejo/wrae193)
Supplement: AdditionalFile2_wrae193 [file additionalfile2_wrae193.docx]

# High-sugar diet leads to loss of beneficial probiotics in housefly larvae guts

Anna Voulgari-Kokota*^1,2^, Francesco Boatta^3^, Ruud Rijkers^3,4^, Bregje Wertheim^1^, Leo W. Beukeboom^1^, Jacintha Ellers^2^, Joana Falcao Salles^1^

^1^ Groningen Institute for Evolutionary Life Sciences (GELIFES), University of Groningen, P.O. Box 11103, 9700 CC, Groningen, The Netherlands

^2^ Laboratory of Microbiology, Wageningen University, 6700 EH, Wageningen, The Netherlands

^3^ Amsterdam Institute for Life and Environment, Section Ecology and Evolution, Vrije Universiteit Amsterdam, 1081 HV, Amsterdam, The Netherlands

^4^ Department of Environmental Science, Stockholm University, SE-106 91, Stockholm, Sweden

*corresponding author: [anna.voulgarikokota@wur.nl](mailto:anna.voulgarikokota@wur.nl)

**Table S1.** Indicative bacterial taxa of the housefly gut microbiota for every experimental diet.

| **larval gut microbiota** | | | |  | **adult gut microbiota** | | | |
| --- | --- | --- | --- | --- | --- | --- | --- | --- |
| **diet: CTR** | | | | | | | | |
| ASV id | indicative taxa | *IndVal* |  |  | ASV id | Indicative taxa | *IndVal* |  |
| 4fed313cc023b70757416818eceb46b8 | *Lacticaseibacillus* | 0.524 | *** |  | b206c3714722b185943200fef42f8a8b | *Leucobacter* | 0.523 | *** |
| fafcda05e3b35ccc8e815e37596fca3f | *Latilactobacillus* | 0.504 | *** |  | 93a4c4a6b2085149869ce2478e32ec87 | *Corynebacterium* | 0.508 | *** |
| 01e8e46dfb17897703df6017d320f0b8 | *Enterococcus* | 0.472 | *** |  | 87cdbd99c098a922fbb03d72c0f0aa61 | *Corynebacterium* | 0.497 | *** |
| 90fc357854594363c85c1ad394513783 | *Enterococcus* | 0.469 | *** |  | d4c03466fd90fc7ff616d83d08791d8d | *Leucobacter* | 0.49 | *** |
| 0568f45cdd2530cd4c1878e6adddde40 | *Latilactobacillus* | 0.463 | *** |  | 66a2f942036ebe4f2878556abe3693b9 | *Leucobacter* | 0.44 | *** |
| 87523b9fd62aef8b8597fd3a3e9e0784 | *Limosilactobacillus* | 0.427 | *** |  | 7396b440ece5270ba23a98a23758ec49 | *Leucobacter* | 0.401 | ** |
| 544104b6eae5aca5f3c86e5cafc87ae9 | *Limosilactobacillus* | 0.424 | *** |  | 648865f96c0d4cc6d336fc9516569d3c | *Myroides* | 0.389 | ** |
| 559c76bdd5e3b9c7bf56913847a79e7c | *Limosilactobacillus* | 0.414 | ** |  | ec144f2727fdfd5b55c975c63a003dbb | *Myroides* | 0.384 | ** |
| b51542b14431ff777879094d739a6a94 | *Lactiplantibacillus* | 0.408 | ** |  | 93566994b6e68ebacba0023263247398 | *Listeria* | 0.373 | ** |
| 886a89160631acdbf9d6b573171b8436 | *Lactiplantibacillus* | 0.402 | ** |  | 3d278dfab94fa8d4ad4264d86d0f7327 | *Sphingobacterium* | 0.336 | * |
| c518c52ce280f3e8ba691e9c746d6722 | Enterobacteriaceae | 0.363 | ** |  |  |  |  |  |
| 8d076c4c83fc126aff80e1f9762a75e4 | *Pediococcus* | 0.357 | ** |  |  |  |  |  |
| 8ab808036be1ae0d31433197d768954f | Enterobacteriaceae | 0.35 | ** |  |  |  |  |  |
| 854d46c40dac7070a8efe689977d3c40 | *Kocuria* | 0.314 | ** |  |  |  |  |  |
| **diet: HF** | | | | | | | | |
| ASV id | indicative taxa | *IndVal* |  |  | ASV id | indicative taxa | *IndVal* |  |
| 40bb8cb9d05183df71955a693eefad76 | *Staphylococcus* | 0.555 | *** |  | 4f07d0fac0a9909741611aec01cfd3cc | *Weissella* | 0.423 | ** |
| 743566a4569ee8e4a9e53fece423ac8f | *Corynebacterium* | 0.549 | *** |  | 259b3450cebe854b371af4c244a9cfdd | *Weissella* | 0.423 | ** |
| debd17036d4bd7b6420cba8c8999bd76 | *Staphylococcus* | 0.546 | *** |  | 05828e8b8011ec77315f89729f5f88fd | *Pediococcus* | 0.403 | ** |
| 8913ac1438638467be0bb85928dabe93 | *Corynebacterium* | 0.533 | *** |  | 671fc139d0e3e8dd95b97d9464babb6d | *Weissella* | 0.377 | ** |
| 470ac330fc294077fd431a6f9084c3d2 | *Staphylococcus* | 0.446 | *** |  | fcb466b217a1c51ba1db56006c4b108f | *Weissella* | 0.37 | ** |
| eb721e4055b8d8398d473758559adc1e | *Enterococcus* | 0.424 | *** |  | 3b51ff954e4ca137be93186608b23d64 | *Pediococcus* | 0.364 | ** |
| 8c66b740cba7210b7da6230582035202 | *Corynebacterium* | 0.382 | ** |  | da0c4881e83a46f33961e13f386a7ad3 | *Serratia* | 0.36 | * |
| f4d85d3176d3c3bd34ff02e4fe8b16f1 | *Staphylococcus* | 0.331 | * |  | 6d2b62ac5e29a88859908b6fcb8eac0f | *Serratia* | 0.36 | * |
| f2845b6205228d61294c1d5c5cc0b75d | *Staphylococcus* | 0.312 | ** |  | 43523c3343d55098ca0b8decc3550e73 | *Pediococcus* | 0.36 | ** |
| dd6cd55585049ac3f72abf871f968dc3 | *Enterococcus* | 0.311 | * |  | 8265f325762ab550a3371254655fa26f | *Reyranella* | 0.352 | ** |
| eb89ef905bbed88b03f48f459f7fd6ff | *Enterococcus* | 0.31 | * |  | 9c4b4ff5e97816c45583774d76630cf8 | *Protochlamydia* | 0.352 | * |
| 1adee8f4703f5f158697d1ca18dd3d97 | *Acinetobacter* | 0.302 | *** |  | 216b37fc97cf27884a541df66c5cd859 | *Coxiella* | 0.349 | * |
|  |  |  |  |  | a3fc3f754460b645a0636717d0c23659 | *Micrococcaceae* | 0.345 | * |
|  |  |  |  |  | b26eb9c9e0df82aceb09edd5044afc8f | *Reyranella* | 0.344 | ** |
|  |  |  |  |  | 93bc3dbf118281ae5f2f01b6f2944919 | *Stenotrophomonas* | 0.335 | ** |
|  |  |  |  |  | e4d6eaee2ad500757d3a71d9142025ec | *Fibrisoma* | 0.333 | ** |
|  |  |  |  |  | aa1087fbdac1d7ddcbf685e132e40982 | *Paludisphaera* | 0.328 | ** |
|  |  |  |  |  | c55cc2d2f61977411faad788b1c2cce4 | *Protochlamydia* | 0.317 | * |
|  |  |  |  |  | f1fef559fe9357f30a5a42dc247cb8ec | *Achromobacter* | 0.306 | * |
| **diet: HS** | | | | | | | | |
| ASV id | indicative taxa | *IndVal* |  |  | ASV id | indicative taxa | *IndVal* |  |
| 259b3450cebe854b371af4c244a9cfdd | *Weissella* | 0.513 | *** |  | 8913ac1438638467be0bb85928dabe93 | *Corynebacterium* | 0.56 | *** |
| 4f07d0fac0a9909741611aec01cfd3cc | *Weissella* | 0.508 | *** |  | 743566a4569ee8e4a9e53fece423ac8f | *Corynebacterium* | 0.553 | *** |
| 2b70263327213fbc057feb48165a6b45 | *Weissella* | 0.498 | *** |  | 42b4ea64a8f9394434431028eff0daa8 | *Brachybacterium* | 0.416 | *** |
| a7fe1ebd4ff08b3211b23cc4578dab73 | Micrococcaceae | 0.492 | *** |  | d911dda8a63f33b3f1ca201bac28e8dc | *Acinetobacter* | 0.362 | ** |
| 3b51ff954e4ca137be93186608b23d64 | *Pediococcus* | 0.432 | *** |  | c833751fcb8fc3ab4d5b7dfc97c55259 | *Brevibacterium* | 0.359 | * |
| 05828e8b8011ec77315f89729f5f88fd | *Pediococcus* | 0.405 | *** |  | 0e9309dab25421f42a82edaaedb0474b | *Bacillus* | 0.344 | * |
| e515cea7b4e909db6e5c0fcc6233a0ed | *Providencia* | 0.371 | ** |  | 594dd12f92909fe1d4ee040a8d8dc6c5 | *Weissella* | 0.343 | * |
| f58275bd620d45ed1584cb78b636ad03 | Micrococcaceae | 0.328 | ** |  | f88ce9c3a9be7bbbc6eeedfb7840a095 | *Weissella* | 0.335 | * |
| c833751fcb8fc3ab4d5b7dfc97c55259 | *Brevibacterium* | 0.317 | ** |  | 07c47db69141b446eaef881f08139a20 | *Lachnoclostridium* | 0.33 | * |
|  |  |  |  |  | 91530be2888d2f6857f47bd877955995 | *Brevibacterium* | 0.319 | * |
|  |  |  |  |  | d36bcac7c612d2186cbd5e8077e8f72a | *Corynebacterium* | 0.316 | * |
|  |  |  |  |  | 995657c32275d4b13619f13baf8ec454 | *Sphingobium* | 0.314 | * |
|  |  |  |  |  | eec975b338ac3108e54f9fdcb640122c | *Enterococcus* | 0.314 | * |
|  |  |  |  |  | 595a2bbf23476033d7e5c7c1ceae589d | *Corynebacterium* | 0.313 | * |
|  |  |  |  |  | dd6cd55585049ac3f72abf871f968dc3 | *Enterococcus* | 0.305 | * |

Indicative bacterial taxa of the housefly gut microbiota for every experimental diet. CTR stands for the control, HF stands for the high-fat and HS stands for the high-sugar larval substrate. The *IndVal* stat value stands for the association strength between each ASV and diet type; values > 0.3 are included. Asterisks indicate significant associations (*P*<0.05*, *P*<0.01**, *P*<0.001***).
