# Supplementary material for: High-sugar diet leads to loss of beneficial probiotics in housefly larvae guts
Source: ISME J. 2024 Oct 3;18(1):wrae193. doi: 10.1093/ismejo/wrae193 (PMC11495414; doi:10.1093/ismejo/wrae193)
Supplement: AdditionalFile3_wrae193 [file additionalfile3_wrae193.docx]

# High-sugar diet leads to loss of beneficial probiotics in housefly larvae guts

Anna Voulgari-Kokota*^1,2^, Francesco Boatta^3^, Ruud Rijkers^3,4^, Bregje Wertheim^1^, Leo W. Beukeboom^1^, Jacintha Ellers^2^, Joana Falcao Salles^1^

^1^ Groningen Institute for Evolutionary Life Sciences (GELIFES), University of Groningen, P.O. Box 11103, 9700 CC, Groningen, The Netherlands

^2^ Laboratory of Microbiology, Wageningen University, 6700 EH, Wageningen, The Netherlands

^3^ Amsterdam Institute for Life and Environment, Section Ecology and Evolution, Vrije Universiteit Amsterdam, 1081 HV, Amsterdam, The Netherlands

^4^ Department of Environmental Science, Stockholm University, SE-106 91, Stockholm, Sweden

*corresponding author: [anna.voulgarikokota@wur.nl](mailto:anna.voulgarikokota@wur.nl)

**Table S2.** Mixed effect models results for the relative abundance of the most prevalent bacterial genera

| **Mixed model** | **Bacterial genus:** | | ***Weissella*** | |
| --- | --- | --- | --- | --- |
| anova(model) | numDF | denDF | F value | *P* value |
| (Intercept) | 1 | 57 | 567.8579 | <.0001 |
| diet | 2 | 12 | 28.8020 | <.0001 |
| generation | 1 | 57 | 41.4380 | <.0001 |
| diet:generation | 2 | 57 | 18.7280 | <.0001 |
|  |  |  |  |  |
| Comparison of models with and without the experimental line as a random effect variable | | | | |
| anova(model.random, model.fixed) | | *P value*=1 | | |
|  | |  |  |  |
| Cox and Snell (ML) | | pseudo R^2^= 0.6518 | | |
| Likelihood ratio test | | *P value* < 0.0001 | | |
|  |  |  |  |  |
| Marginal model categories | | | | |
| CTR | a |  |  |  |
| HF | a |  |  |  |
| HS | b |  |  |  |
| **Mixed model** | **Bacterial genus:** | | ***Lactiplantibacillus*** | |
| anova(model)v | numDF | denDF | F value | *P value* |
| (Intercept) | 1 | 57 | 99.8150 | <.0001 |
| diet | 2 | 12 | 31.0913 | <.0001 |
| generation | 1 | 57 | 98.4329 | <.0001 |
| diet:generation | 2 | 57 | 42.8044 | <.0001 |
|  |  |  |  |  |
| Comparison of models with and without the experimental line as a random effect variable | | | | |
| anova(model.random, model.fixed) | | *P value*=1 | | |
|  | |  |  |  |
| Cox and Snell (ML) | | pseudo R^2^= 0.781147 | | |
| Likelihood ratio test | | *P value* < 0.0001 | | |
|  |  |  |  |  |
| Marginal model categories | | | | |
| CTR | c |  |  |  |
| HF | b |  |  |  |
| HS | a |  |  |  |
| **Mixed model** | **Bacterial genus:** | | ***Limosilactobacillus*** | |
| anova(model) | numDF | denDF | F value | *P value* |
| (Intercept) | 1 | 57 | 255.2065 | <.0001 |
| diet | 2 | 12 | 56.5478 | <.0001 |
| generation | 1 | 57 | 219.3845 | <.0001 |
| diet:generation | 2 | 57 | 59.3799 | <.0001 |
|  |  |  |  |  |
| Comparison of models with and without the experimental line as a random effect variable | | | | |
| anova(model.random, model.fixed) | | *P value*=1 | | |
|  | |  |  |  |
| Cox and Snell (ML) | | pseudo R^2^= 0.867098 | | |
| Likelihood ratio test | | *P value* < 0.0001 | | |
|  |  |  |  |  |
| Marginal model categories | | | | |
| CTR | c |  |  |  |
| HF | b |  |  |  |
| HS | a |  |  |  |
| **Mixed model** | **Bacterial genus:** | | ***Pediococcus*** | |
| anova(model) | numDF | denDF | F value | *P value* |
| (Intercept) | 1 | 57 | 258.1477 | <.0001 |
| diet | 2 | 12 | 12.1183 | 0.0013 |
| generation | 1 | 57 | 23.6847 | <.0001 |
| diet:generation | 2 | 57 | 10.44621 | <.0001 |
|  |  |  |  |  |
| Comparison of models with and without the experimental line as a random effect variable | | | | |
| anova(model.random, model.fixed) | | *P value*=1 | | |
|  | |  |  |  |
| Cox and Snell (ML) | | pseudo R^2^= 0.499389 | | |
| Likelihood ratio test | | *P value* < 0.0001 | | |
|  | |  |  |  |
| Marginal model categories | | | | |
| CTR | a |  |  |  |
| HF | a |  |  |  |
| HS | b |  |  |  |
| **Mixed model** | **Bacterial genus:** | | ***Providencia*** | |
| anova(model) | numDF | denDF | F value | *P value* |
| (Intercept) | 1 | 57 | 165.9084 | <.0001 |
| diet | 2 | 12 | 5.9396 | 0.0161 |
| generation | 1 | 57 | 0.8543 | 0.3592 |
| diet:generation | 2 | 57 | 3.8284 | 0.0275 |
|  |  |  |  |  |
| Comparison of models with and without the experimental line as a random effect variable | | | | |
| anova(model.random, model.fixed) | | *P value*=1 | | |
|  | |  |  |  |
| Cox and Snell (ML) | | pseudo R^2^= 0.218896 | | |
| Likelihood ratio test | | *P value*= 0.0050386 | | |
|  |  |  |  |  |
| Marginal model categories | | | | |
| CTR | ab |  |  |  |
| HF | a |  |  |  |
| HS | b |  |  |  |
| **Mixed model** | **Bacterial genus:** | | ***Corynebacterium*** | |
| anova(model) | numDF | denDF | F value | *P value* |
| (Intercept) | 1 | 57 | 211.6547 | <.0001 |
| diet | 2 | 12 | 22.9938 | <.0001 |
| generation | 1 | 57 | 0.4405 | 0.5096 |
| diet:generation | 2 | 57 | 5.5482 | 0.0063 |
|  |  |  |  |  |
| Comparison of models with and without the experimental line as a random effect variable | | | | |
| anova(model.random, model.fixed) | | *P value*=1 | | |
|  | |  |  |  |
| Cox and Snell (ML) | | pseudo R^2^= 0.402507 | | |
| Likelihood ratio test | | *P value* < 0.0001 | | |
|  |  |  |  |  |
| Marginal model categories | | | | |
| CTR | b |  |  |  |
| HF | c |  |  |  |
| HS | a |  |  |  |
| **Mixed model** | **Bacterial genus:** | | ***Morganella*** | |
| anova(model) | numDF | denDF | F value | *P value* |
| (Intercept) | 1 | 57 | 35.6126 | <.0001 |
| diet | 2 | 12 | 1.1959 | 0.3361 |
| generation | 1 | 57 | 19.1838 | <.0001 |
| diet:generation | 2 | 57 | 0.9791 | 0.3819 |
|  |  |  |  |  |
| Comparison of models with and without the experimental line as a random effect variable | | | | |
| anova(model.random, model.fixed) | | *P value*=1 | | |
|  | |  |  |  |
| Cox and Snell (ML) | | pseudo R^2^= 0.254326 | | |
| Likelihood ratio test | | *P value* = 0.0012058 | | |
|  |  |  |  |  |
| Marginal model categories | | | | |
| CTR | a |  |  |  |
| HF | a |  |  |  |
| HS | a |  |  |  |
| **Mixed model** | **Bacterial genus:** | | ***Myroides*** | |
| anova(model) | numDF | denDF | F value | *P value* |
| (Intercept) | 1 | 57 | 6.3062 | 0.0149 |
| diet | 2 | 12 | 3.2472 | 0.0746 |
| generation | 1 | 57 | 2.9005 | 0.0940 |
| diet:generation | 2 | 57 | 2.9005 | 0.0632 |
|  |  |  |  |  |
| Comparison of models with and without the experimental line as a random effect variable | | | | |
| anova(model.random, model.fixed) | | *P value*=1 | | |
|  | |  |  |  |
| Cox and Snell (ML) | | pseudo R^2^= 0.651814 | | |
| Likelihood ratio test | | *P value* =0.020826 | | |
|  |  |  |  |  |
| Marginal model categories | | | | |
| CTR | a |  |  |  |
| HF | a |  |  |  |
| HS | a |  |  |  |
| **Mixed model** | **Bacterial genus:** | | ***Proteus*** | |
| anova(model) | numDF | denDF | F value | *P value* |
| (Intercept) | 1 | 57 | 9.7780 | 0.0028 |
| diet | 2 | 12 | 3.5089 | 0.0631 |
| generation | 1 | 57 | 7.7511 | 0.0073 |
| diet:generation | 2 | 57 | 3.0437 | 0.0555 |
|  |  |  |  |  |
| Comparison of models with and without the experimental line as a random effect variable | | | | |
| anova(model.random, model.fixed) | | *P value*=1 | | |
|  | |  |  |  |
| Cox and Snell (ML) | | pseudo R^2^= 0.232107 | | |
| Likelihood ratio test | | *P value* = 0.0029961 | | |
|  |  |  |  |  |
| Marginal model categories | | | | |
| CTR | a |  |  |  |
| HF | a |  |  |  |
| HS | a |  |  |  |
| **Mixed model** | **Bacterial genus:** | | ***Lactococcus*** | |
| anova(model) | numDF | denDF | F value | *P value* |
| (Intercept) | 1 | 57 | 21.6247 | <.0001 |
| diet | 2 | 12 | 0.6655 | 0.5320 |
| generation | 1 | 57 | 7.3117 | 0.0090 |
| diet:generation | 2 | 57 | 0.5907 | 0.5573 |
|  |  |  |  |  |
| Comparison of models with and without the experimental line as a random effect variable | | | | |
| anova(model.random, model.fixed) | | *P value*=1 | | |
|  | |  |  |  |
| Cox and Snell (ML) | | pseudo R^2^= 0.124634 | | |
| Likelihood ratio test | | *P value* = 0.12535 | | |
|  |  |  |  |  |
| Marginal model categories | | | | |
| CTR | a |  |  |  |
| HF | a |  |  |  |
| HS | a |  |  |  |
| **Mixed model** | **Bacterial genus:** | | ***Acinetobacter*** | |
| anova(model) | numDF | denDF | F value | *P value* |
| (Intercept) | 1 | 57 | 9.1725 | 0.0037 |
| diet | 2 | 12 | 7.4426 | 0.0079 |
| generation | 1 | 57 | 14.1976 | 0.0004 |
| diet:generation | 2 | 57 | 11.9336 | <.0001 |
|  |  |  |  |  |
| Comparison of models with and without the experimental line as a random effect variable | | | | |
| anova(model.random, model.fixed) | | *P value*= 0.9984 | | |
|  | |  |  |  |
| Cox and Snell (ML) | | pseudo R^2^ =0.433701 | | |
| Likelihood ratio test | | *P value* < 0.0001 | | |
|  |  |  |  |  |
| Marginal model categories | | | | |
| CTR | a |  |  |  |
| HF | b |  |  |  |
| HS | a |  |  |  |
| **Mixed model** | **Bacterial genus:** | | ***Latilactobacillus*** | |
| anova(model) | numDF | denDF | F value | *P value* |
| (Intercept) | 1 | 57 | 50.3527 | <.0001 |
| diet | 2 | 12 | 12.6950 | 0.0011 |
| generation | 1 | 57 | 1.1552 | 0.2870 |
| diet:generation | 2 | 57 | 0.5633 | 0.5725 |
|  |  |  |  |  |
| Comparison of models with and without the experimental line as a random effect variable | | | | |
| anova(model.random, model.fixed) | | *P value*=1 | | |
|  | |  |  |  |
| Cox and Snell (ML) | | pseudo R^2^= 0.264798 | | |
| Likelihood ratio test | | *P value* = 0.00077312 | | |
|  |  |  |  |  |
| Marginal model categories | | | | |
| CTR | b |  |  |  |
| HF | a |  |  |  |
| HS | a |  |  |  |
| **Mixed model** | **Bacterial genus:** | | ***Enterococcus*** | |
| anova(model) | numDF | denDF | F value | *P value* |
| (Intercept) | 1 | 57 | 118.8335 | <.0001 |
| diet | 2 | 12 | 23.1007 | <.0001 |
| generation | 1 | 57 | 34.2623 | <.0001 |
| diet:generation | 2 | 57 | 8.1047 | 0.0008 |
|  |  |  |  |  |
| Comparison of models with and without the experimental line as a random effect variable | | | | |
| anova(model.random, model.fixed) | | *P value*=0.9789 | | |
|  | |  |  |  |
| Cox and Snell (ML) | | pseudo R^2^= 0.560112 | | |
| Likelihood ratio test | | *P value* < 0.0001 | | |
|  |  |  |  |  |
| Marginal model categories | | | | |
| CTR | c |  |  |  |
| HF | b |  |  |  |
| HS | a |  |  |  |
| **Mixed model** | **Bacterial genus:** | | ***Staphylococcus*** | |
| anova(model) | numDF | denDF | F value | *P value* |
| (Intercept) | 1 | 57 | 46.7211 | <.0001 |
| diet | 2 | 12 | 16.7435 | 0.0003 |
| generation | 1 | 57 | 22.5991 | <.0001 |
| diet:generation | 2 | 57 | 8.0984 | 0.0008 |
|  |  |  |  |  |
| Comparison of models with and without the experimental line as a random effect variable | | | | |
| anova(model.random, model.fixed) | | *P value*=1 | | |
|  | |  |  |  |
| Cox and Snell (ML) | | pseudo R^2^= 0. 498535 | | |
| Likelihood ratio test | | *P value* < 0.0001 | | |
|  |  |  |  |  |
| Marginal model categories | | | | |
| CTR | a |  |  |  |
| HF | b |  |  |  |
| HS | a |  |  |  |
| **Mixed model** | **Bacterial genus:** | | ***Lacticaseibacillus*** | |
| anova(model) | numDF | denDF | F value | *P value* |
| (Intercept) | 1 | 57 | 49.2973 | <.0001 |
| diet | 2 | 12 | 49.2973 | <.0001 |
| generation | 1 | 57 | 63.9828 | <.0001 |
| diet:generation | 2 | 57 | 63.9828 | <.0001 |
|  |  |  |  |  |
| Comparison of models with and without the experimental line as a random effect variable | | | | |
| anova(model.random, model.fixed) | | *P value*=1 | | |
|  | |  |  |  |
| Cox and Snell (ML) | | pseudo R^2^= 0.804430 | | |
| Likelihood ratio test | | *P value* < 0.0001 | | |
|  |  |  |  |  |
| Marginal model categories | | | | |
| CTR | b |  |  |  |
| HF | a |  |  |  |
| HS | a |  |  |  |

Mixed effect models results for the relative abundance of the most prevalent bacterial genera found in the housefly larval microbiota, through the course of 13 generations. The occurrence of the bacterial genera measured at each generation was treated as repetitive measuring. Diet and generation were set as fixed factors. The housefly experimental line was either included or not included as a random factor to assess its effect. In the end, each diet is set at a level (category), according the occurrence of each genus.
