# Supplementary material for: High-sugar diet leads to loss of beneficial probiotics in housefly larvae guts
Source: ISME J. 2024 Oct 3;18(1):wrae193. doi: 10.1093/ismejo/wrae193 (PMC11495414; doi:10.1093/ismejo/wrae193)
Supplement: Additional_File_wrae193 [file additional_file_wrae193.docx]

AdditionalFile1.docx: Supporting figures referenced in the main manuscript.

AdditionalFile2.docx: Supporting table with indicative bacterial taxa of the housefly gut microbiota for every experimental diet

AdditionalFile3.docx: Supporting table with mixed effect models results for the relative abundance of the most prevalent bacterial genera

AdditionalFile4.xsl: Alignment and phylogeny of ASVs assigned as *Weissella*, *Lactiniplantibacillus* and *Limosilactobacillus* with the 16S rRNA sequences from three isolated strains.

AdditionalFile5.xsl: Relative abundance of metabolic pathways of microbiota in samples of housefly larvae reared on three experimental diets with and without probiotics
